# Omp2b Porin Alteration in the Course of Evolution of *Brucella* spp.

**DOI:** 10.3389/fmicb.2020.00284

**Published:** 2020-02-24

**Authors:** Axel Cloeckaert, Gilles Vergnaud, Michel S. Zygmunt

**Affiliations:** ^1^INRAE, UMR ISP, Université de Tours, Nouzilly, France; ^2^Institute for Integrative Biology of the Cell (I2BC), CEA, CNRS, Université Paris-Saclay, Gif-sur-Yvette, France

**Keywords:** *Brucella*, Omp2 porin, loop, diversity, gene conversion, evolution

## Abstract

The genus *Brucella* comprises major pathogenic species causing disease in livestock and humans, e.g. *B. melitensis*. In the past few years, the genus has been significantly expanded by the discovery of phylogenetically more distant lineages comprising strains from diverse wildlife animal species, including amphibians and fish. The strains represent several potential new species, with *B. inopinata* as solely named representative. Being genetically more distant between each other, relative to the “classical” *Brucella* species, they present distinct atypical phenotypes and surface antigens. Among surface protein antigens, the Omp2a and Omp2b porins display the highest diversity in the classical *Brucella* species. The genes coding for these proteins are closely linked in the *Brucella* genome and oriented in opposite directions. They share between 85 and 100% sequence identity depending on the *Brucella* species, biovar, or genotype. Only the *omp2b* gene copy has been shown to be expressed and genetic variation is extensively generated by gene conversion between the two copies. In this study, we analyzed the *omp2* loci of the non-classical *Brucella* spp. Starting from two distinct ancestral genes, represented by Australian rodent strains and *B. inopinata*, a stepwise nucleotide reduction was observed in the *omp2b* gene copy. It consisted of a first reduction affecting the region encoding the surface L5 loop of the porin, previously shown to be critical in sugar permeability, followed by a nucleotide reduction in the surface L8 loop-encoding region. It resulted in a final *omp2b* gene size shared between two distinct clades of non-classical *Brucella* spp. (African bullfrog isolates) and the group of classical *Brucella* species. Further evolution led to complete homogenization of both *omp2* gene copies in some *Brucella* species such as *B. vulpis* or *B. papionis*. The stepwise *omp2b* deletions seemed to be generated through recombination with the respective *omp2a* gene copy, presenting a conserved size among *Brucella* spp., and may involve short direct DNA repeats. Successive Omp2b porin alteration correlated with increasing porin permeability in the course of evolution of *Brucella* spp. They possibly have adapted their porin to survive environmental conditions encountered and to reach their final status as intracellular pathogen.

## Introduction

Members of the genus *Brucella* are Gram-negative, facultative, intracellular bacteria that can infect many species of animals and man. Until the 1990s, six species were classically recognized within the genus *Brucella*: *B. abortus*, *B. melitensis*, *B. suis*, *B. ovis*, *B. canis*, and *B. neotomae* (Corbel and Brinley Morgan, 1984; Moreno et al., 2002; Godfroid et al., 2011). This classification was mainly based on differences in pathogenicity, host preference, and phenotypic characteristics (Alton et al., 1988). Since then, with the help of modern molecular typing methods [e.g. Multiple Loci Sequence Analysis (MLSA) and Multiple Loci VNTR Analysis (MLVA)] and whole genome sequencing (WGS), a number of new species representing mostly wildlife isolates and showing very different phenotypes have been validly published. In chronological order it concerns the species (i) *B. ceti* and *B. pinnipedialis* isolated from marine mammals, with cetaceans (dolphin, porpoise, and whale species) and pinnipeds (various seal species) as preferred hosts, respectively (Foster et al., 2007); (ii) *B. microti* isolated initially from the common vole but found later also in red foxes, in soil, and most recently in marsh frogs (Scholz et al., 2008a, b, 2009; Jaÿ et al., 2018); (iii) *B. inopinata* isolated from human (Scholz et al., 2010); (iv) *B. papionis* isolated from baboons (Whatmore et al., 2014); and (v) the latest *B. vulpis* species isolated from red foxes (Scholz et al., 2016a). Novel *Brucella* strains representing potentially novel species have also been isolated from Australian rodents (Tiller et al., 2010a), a wide variety of frog species (Eisenberg et al., 2012; Fischer et al., 2012; Scholz et al., 2016b; Soler-Lloréns et al., 2016; Al Dahouk et al., 2017; Kimura et al., 2017; Mühldorfer et al., 2017), and surprisingly also from fish namely from a bluespotted ribbontail ray (*Taeniura lymma*) (Eisenberg et al., 2017). The genus *Brucella* nowadays is thus not restricted to mammal species. The most recent potential new species reported is isolated from a dog in Costa Rica in the early 1980s (Guzmán-Verri et al., 2019).

*Brucella* spp. consist nowadays of two major groups. The first represents the species termed “classical” and consists of the six initially recognized species together with the more recent species *B. ceti*, *B. pinnipedialis*, *B. microti*, and *B. papionis*. The further subdivision in MLSA or MLVA genotypes proved to be helpful for subtyping of some species. For example *B. ceti* genotype ST23 is currently mainly found in several cetacean species (porpoise, dolphin, whale), whereas *B. ceti* genotype ST26 seems more restricted to dolphin species (Maquart et al., 2009; Whatmore et al., 2016; Vergnaud et al., 2018). The first group has been characterized and investigated in detail because of its importance in causing disease in animals and humans. The second major group is represented by lineages phylogenetically more distant from the classical species and still poorly characterized. This group comprises several distinct subgroups consisting of isolates from diverse wildlife animal species cited above, i.e. Red foxes, Australian rodents, several frog species, the isolate from a ray fish, and interestingly also two isolates from human cases. These subgroups represent several potential new species, with *B. inopinata*, isolated from a human case, as solely named representative. Being genetically more distant between each other, relative to the classical *Brucella* species, they present distinct atypical phenotypes and surface antigens. For example several amphibian subgroups and the human isolate *Brucella* sp. BO2 present distinct unidentified O antigens, that are not typable by polyclonal or monoclonal antibodies used for serotyping of the classical species (Tiller et al., 2010b; Zygmunt et al., 2012; Al Dahouk et al., 2017).

Among outer membrane proteins, the Omp2 porins have been shown to display the highest diversity within the classical *Brucella* species, which allowed to differentiate them at the species, biovar, or genotype level (Ficht et al., 1990, 1996; Cloeckaert et al., 1995, 2001). More precisely, the porins are encoded by the *omp2* locus, consisting of two closely related genes *omp2a* and *omp2b*, separated by approximately 830 bp and oriented in opposite directions (Ficht et al., 1989; Marquis and Ficht, 1993). Gene diversity is extensively generated by recombination between both copies called also gene conversion (Santoyo and Romero, 2005), thus resulting in *omp2a* and *omp2b* gene copies sharing from 82 to 100% nucleotide identity depending on the species, biovar or genotype. The highest divergence between the gene copies in classical species was found in *B. microti* and *B. melitensis* (82.4 and 83.4% identity, respectively, see Supplementary Figure S1). In contrast, complete homogenization of the copies (100% identity) was observed for *B. ceti* ST26 or *B. papionis* (Supplementary Figure S1). Several other situations may be observed and in former studies *Brucella* strains were sometimes described in a simple way as carrying either (i) both the *omp2a* and *omp2b* gene copies (e.g. *B. melitensis*), (ii) two *omp2a* gene copies (e.g. *B. ovis*), or (iii) two *omp2b* gene copies (e.g. *B. ceti*) (Ficht et al., 1990, 1996; Cloeckaert et al., 2001). The release of additional sequences showed that the situation is more complex because of the existence of numerous intermediate situations, including also *B. ovis* whose gene copies are actually not 100% *omp2a* identical. Only the *omp2b* gene copy has been shown to be expressed (Marquis and Ficht, 1993), and the genetic exchanges that may occur with its *omp2a* counterpart may affect the sugar permeability of the expressed porin as previously shown for naturally chimeric Omp2a and Omp2b porin variants (Paquet et al., 2001). The evaluated Omp2a variant showed a more efficient pore in sugar diffusion than the Omp2b variant. Differences in their respective L5 surface-exposed loop were suggested to play a major role in permeability, the L5 loop of Omp2a being shorter and containing less negatively charged residues than that of Omp2b.

Porins being important first line players to resist and adapt to environmental conditions, in the present study we analyzed the *omp2* loci and their encoded porins from non-classical *Brucella* spp. to identify possible evolutionary paths that may have contributed to the adaptation to their final status as intracellular mammal or human pathogens.

## Materials and Methods

*Brucella* strains analyzed were from previous studies (Scholz et al., 2010, 2016a; Tiller et al., 2010a, b; Zygmunt et al., 2012; Al Dahouk et al., 2017; Eisenberg et al., 2017), and are listed in the Supplementary Figure S1. Bacterial cultures and DNA extraction were performed as described previously (Cloeckaert et al., 1995). For the African bullfrog isolates of this study, DNA was extracted from killed bacterial cells provided by Dr. S. Al Dahouk (BfR, Berlin, Germany). PCR amplification of the *omp2a* and *omp2b* genes was performed as described previously (Cloeckaert et al., 1995, 2001). The PCR products were sequenced at Genome Express (Meylan, France). Nucleotide sequence analysis and alignments were done using Clustal Omega at EBI^1^. GenBank nucleotide accession numbers are indicated in Supplementary Figure S1. *omp2* sequences were aligned using BioNumerics version 7.6.3 (Applied Maths, Belgium) with default parameters and the multiple alignment was used to produce a Maximum Parsimony tree. Comparative Omp2 protein analyses were done using a previously published Omp2a and Omp2b predicted and functionally validated topology model (Paquet et al., 2001).

Whole genome SNP (wgSNP) analysis was done as previously described (Vergnaud et al., 2018). Full genome sequences and assemblies were converted into artificial reads. SNPs were identified by mapping sequencing reads on a reference genome within BioNumerics. Assembly GCF_000007125 (*B. melitensis* 16 M) was used as reference. The Bio-Neighbor joining algorithm with bootstrap analysis embedded in BioNumerics was used for clustering analysis (Gascuel, 1997).

## Results and Discussion

### Global View of *omp2* Gene Diversity in *Brucella* spp.

**FIGURE 1:**
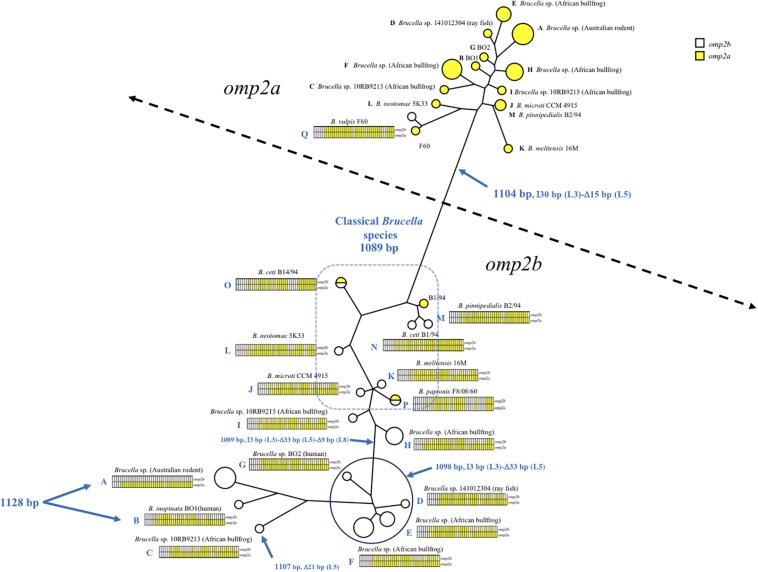
*Brucella* spp. *omp2* clustering analysis, constructed using the *omp2a* and *omp2b* sequences of the non-classical and classical *Brucella* species of this study (GenBank accession numbers are in Supplementary Figure S1). The strains are represented by circles, increasing in size for strains with identical sequences. Empty circles represent the *omp2b* sequences while yellow-colored circles represent the *omp2a* sequences of the respective strains. Two half-colored circles are present in the tree for strains carrying identical *omp2a* and *omp2b* sequences, i.e. *B. papionis* F8/08/60 and *B. ceti* B14/94. The gene conversion events for each strain or groups of strains are schematized by sliced boxes representing *omp2b* (upper part) and *omp2a* (lower part), colored in yellow when *omp2a*-specific sequences are present. Relevant *omp2* size variations are indicated in blue, with the Greek I and D letters meaning insertion and deletion, respectively. The size in bp of each event, as well as the encoded segment concerned (L3, L5, and L8) are indicated.

**FIGURE 2:**
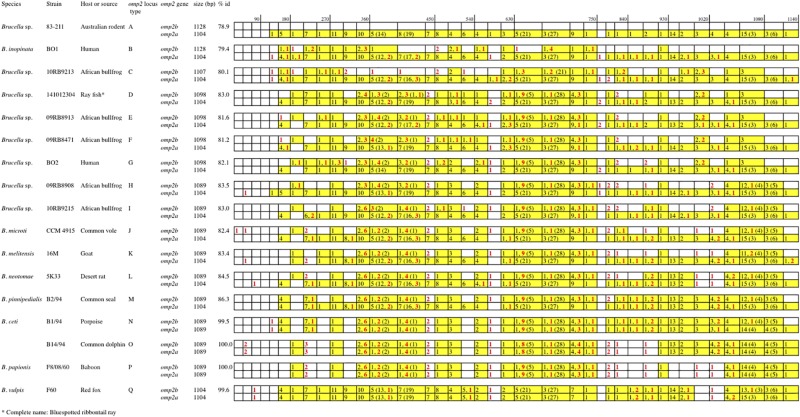
Schematic multiple nucleotide sequence alignment of the *omp2a* and *omp2b* genes of *Brucella* strains. This schematic representation was done according to the detailed alignment show in the Supplementary Figure S1. Nucleotide sequences are represented by rectangles divided into boxes of 30 nucleotides. The *Brucella* sp. 83–211 *omp2b* gene sequence was used as a reference (white boxes). The boxes containing *omp2a*-specific nucleotides are colored in yellow. The numbers in the corresponding boxes indicate the number of *omp2a*-specific nucleotides present in the sequence considered. Numbers in parentheses represent insertions and deletions. Numbers in red indicate nucleotide differences that are not due to gene conversion.

**FIGURE 3:**
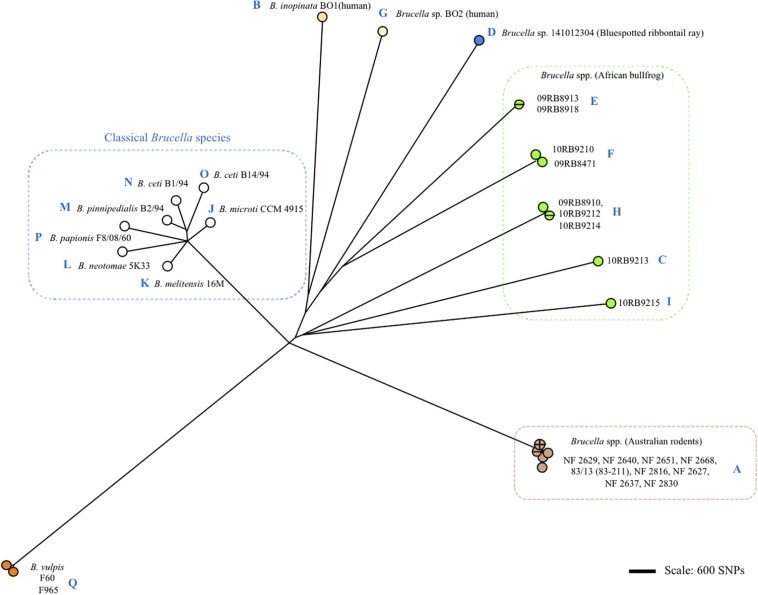
Clustering of *Brucella* spp. and representative classical strains of this study by whole genome SNP calling and Bio-NJ analysis. The color code reflects animal or human host assignment. Strain names are indicated (strain 83/13 is an alias of 83–211). Blue letters A to Q indicate the corresponding *omp2* locus type according to Figure 2 and Supplementary Figure S1.

As shown in Figures 1, 2 and Supplementary Figure S1, *omp2* sequence diversity analysis allowed to separate the non-classical *Brucella* spp. of this study in nine distinct groups (A to I), that correlated perfectly well with other previously published molecular methods such as MLSA, MLVA, IS*711* profiling, or whole genome phylogeny (Figure 3). A second observation was a relative lower nucleotide sequence identity between their respective *omp2a* and *omp2b* gene copy ranging from 78.9 to 83.5%, in comparison to those of classical *Brucella* species ranging from 82.4 to 100% (Figure 2 and Supplementary Figure S1). Considering all *omp2* sequences from both classical and non-classical *Brucella* spp., two separate clusters were observed in the clustering analysis. The “*omp2a*” cluster appeared less diverse than the “*omp2b*” cluster (Figure 1). All *omp2a* representatives shared the same size (1104 bp). In contrast, the size of *omp2b* representatives varied from 1089 to 1128 bp. To analyze in more detail the molecular basis behind this diversity, in particular recombination events, we have used the two most divergent *omp2* gene copies, namely those of *Brucella* spp. isolated from Australian rodents, to designate them as either reference *omp2a* and reference *omp2b* gene copy. It actually provided a more comprehensive evolutionary picture, establishing a clearer link between classical and non-classical strains, than if the most divergent gene copies observed in the classical species only had been used as in previous studies (eg. *B. melitensis* or *B. microti*) (Cloeckaert et al., 2001; Al Dahouk et al., 2012). It also allowed to explain *omp2b* gene size variation, through stepwise genetic reduction, from the size observed for Australian rodent strains to that of the classical *Brucella* species. Starting from these designated reference *omp2a* and *omp2b* gene copies, the effect of genetic recombination events was observed in all groups of strains. Recombination events are schematized in Figure 1 by boxes containing either uncolored segments (*omp2b*) or yellow-colored segments (containing *omp2a*-specific nucleotides) along the groups of strains studied, and these genetic changes are more detailed in Figure 2. The events observed consisted mostly of converting some specific *omp2b* regions into *omp2a*, but the opposite situation (converting *omp2a* regions to *omp2b*) also occurred for some genotypes, resulting in almost complete (e.g. *B. vulpis* or *B. ceti* ST23) or complete (e.g. *B. ceti* ST26 or *B. papionis*) homogenization of both gene copies. Of note is that homogenization did not involve the same *omp2* regions for the examples cited above (see Figure 2), resulting in the placement of the homogenized copies in either the *omp2a* or the *omp2b* cluster of the *omp2* clustering analysis, for, respectively, *B. vulpis* and the other species/genotypes (Figure 1).

Regarding *omp2b* gene size variation, departing from a 1128 bp-sized *omp2b* gene as represented in Australian rodent *Brucella* spp. (group A) and the human *B. inopinata* BO1 isolate (group B), a number of consecutive indel events were observed including: (i) a deletion of 21 bp in the surface-encoding loop L5 in *Brucella* sp. 10RB9213 isolated from an African bullfrog; (ii) a deletion of 33 bp in the same region in four groups of isolates representing two groups from African bullfrogs (groups E and F), one isolate from ray fish (strain 141012304, group D) and the human isolate BO2 (group G); and (iii) and a 9 bp deletion in the region encoding the L8 surface-exposed loop was detected in two groups of African bullfrog strains (H and I), to reach the *omp2b* size of classical species (1089 bp). Those latter African bullfrog *Brucella* spp. thus present the same *omp2b* size as the classical species represented in Figure 1 (from *B. microti* to the marine mammal species *B. ceti* and *B. pinnipedialis*).

Relative to *omp2b* of classical species (1089 bp), the 1104 bp-sized *omp2a* differs by an insertion of 30 bp in the region encoding the L3 surface loop and a deletion of 15 bp in the region encoding the L5 surface loop (Figure 1). The conserved size of the *omp2a* gene copy in both the non-classical and classical species suggests that, as previously observed for classical species (Ficht et al., 1996; Cloeckaert et al., 2002), this gene copy may have been used to convert its *omp2b* counterpart and thus generate the successive *omp2b* gene deletions in the course of evolution of the non-classical species.

### Porin Evolution in the Global Phylogenomic Background Diversity of *Brucella* spp.

Although the Omp2b diversity generated through gene conversion appeared sequential, it did not correlate with the global phylogeny of *Brucella* spp. as shown in Figure 3. More precisely, we did not observe a clear sequential evolutionary link among the non-classical species which appeared genetically far more distant between each other than is reflected by the diversity of their *omp2* locus. It may be explained by the essential functional nature of the Omp2b porin (Laloux et al., 2010), that actually would allow only variation depending on the external or intracellular environmental pressure and favor convergent evolution. In addition, although some SNPs were observed between *omp2* sequences (indicated in Figures and Supplementary Figures S1–S3), Omp2b variation relied mainly on gene conversion and not on SNP variation as for the whole phylogeny of *Brucella* spp.

### Genetic Loss and Functional Alteration of the Omp2b Porin

**FIGURE 4:**
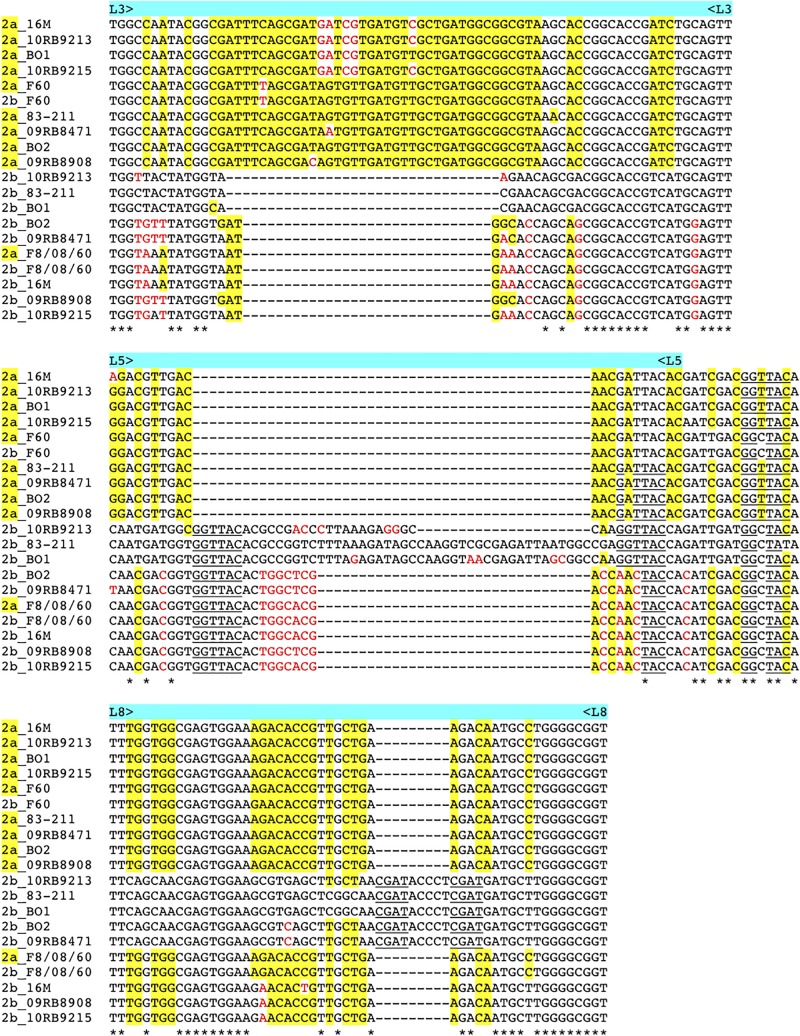
Multiple nucleotide sequence alignment of the *omp2* segments encoding the L3, L5, and L8 loops (upper, middle, and lower panel, respectively), from representative strains containing insertions or deletions. Strains and *omp2* genes used are indicated on the left of each segment, *omp2a* genes are indicated as 2a in yellow. *omp2a*-specific nucleotides are highlighted in yellow, according to the *omp2a* and *omp2b* reference sequences used from *Brucella* sp. 83–211. Nucleotides colored in red indicate differences that are not due to gene conversion, according to the same reference sequences. Direct DNA repeats are underlined. *Indicates identical nucleotides.

**FIGURE 5:**
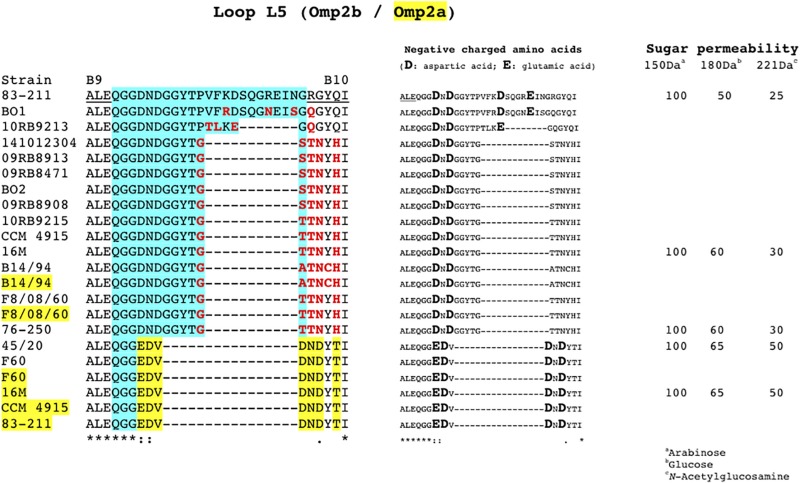
Amino acid sequence alignment of the Omp2b and Omp2a L5 loops from representative strains of this study, showing the progressive loss of amino acids in accordance with the respective nucleotide sequence data shown in Figures 1–3. The strains are indicated on the left, when Omp2a is used the corresponding strain number is highlighted in yellow. The predicted L5 loop, according to Paquet et al. (2001), is highlighted in blue. Underlined B9 and B10 sequences indicate the predicted flanking transmembrane β-strands (Paquet et al., 2001). Omp2a-specific amino acids are highlighted in yellow, in accordance with the Omp2a and Omp2b reference sequence used from *Brucella* sp. 83–211. Omp2 amino acids colored in red are independent from gene conversion according to the same reference sequences. The middle panel of the figure highlights the negatively charged amino acids of the segment. The right panel indicate the available sugar permeability data from previously published strains (Paquet et al., 2001). *Indicates identical amino acids.

As described above *omp2a* or *omp2b* insertions or deletions were predominantly located in regions encoding three predicted surface exposed loops, namely L3, L5, and L8 (Paquet et al., 2001). Figure 4 shows a nucleotide sequence alignment of each region of representative strains of this study, complete sequence alignment is shown in Supplementary Figure S2. The L3 encoding region is characterized by either a 33 bp or 30 bp deletion in *omp2b* relative to *omp2a* in *omp2* groups A, B, and C *Brucella* strains and the other groups, respectively. This 3 bp difference is difficult to explain according to the sequence alignment, but nevertheless in the shorter deletion, *omp2a*-specific sequence fragments remain suggesting that the deletion in *omp2b* resulted from an intra-molecular recombination event between *omp2a* and *omp2b* for this specific region. The L3 loop has been shown to play a major role as constriction loop in most bacterial porins (Jap and Walian, 1996). However, in *Brucella* spp. this loop did not appear critical for sugar permeability according to a previous study (Paquet et al., 2001). According to the same study the L5 loop appeared to be a critical determinant in *Brucella* porin sugar permeability and could participate in the formation of the pore “external mouth,” which serves to prescreen the solute in porins of known structure (Paquet et al., 2001). Consistent with this, two deletions, a first of 21 bp and the second of 33 bp, in the L5 encoding region of the Omp2b porin were identified in the present study, starting from the *omp2* groups A and B strains to reach a minimal L5 size in the other groups of strains, with J to O strains representing the classical *Brucella* species (Figures 1, 4). In addition, it must be noted that *omp2b* from *B. vulpis* (group P in Figures 1, 2) represents a separate situation as its *omp2b* copy has converted to *omp2a* in the L5 region with a concomitant additional 15 bp deletion relative to other *omp2b* sequences (Figure 4), thus making it the shortest Omp2b encoding L5 region of strains of this study. A similar situation has been reported previously for *B. abortus* strain 45/20 for which the same *omp2b* to *omp2a* sequence conversion was observed in this segment (Paquet et al., 2001). The different L5 situations are detailed in the nucleotide sequence alignment of Figure 4. In addition to segmental gene conversion-mediated exchanges between the respective *omp2a* and *omp2b* gene copy, short 6 bp direct DNA repeats may have contributed to generate the different deletions observed in the L5-encoding segment. At the amino acid sequence level it resulted in the loss of either 7 or 11 amino acids including one or two negatively charged residues, respectively (Figure 5), that may possibly be important on ion conductance and ion selectivity of the L5 loop (Paquet et al., 2001). According to previous data, the sequential loss of amino acids observed correlates with increased sugar permeability to reach the highest permeation rate for porin variants containing the shortest Omp2a-type L5 loop (Figure 5; Paquet et al., 2001).

The last genetic reduction observed in the sequential course of Omp2b porin evolution shown in Figure 1, consisted of a 9 bp deletion in the region encoding the surface L8 loop, and it finally ended up to the size of *omp2b* and the respective Omp2b porin protein observed for the classical species. As shown in Figure 5 this short deletion, as observed for the L5 segment, may be the result of gene conversion using the respective *omp2a* copy as template, and two short 4 bp direct repeats may also have contributed to create this deletion. In addition it created an *omp2a*-like segment at the 3′ end of the respective *omp2b* sequence, which did not appear as clearly when using as references *omp2a* and *omp2b* genes from classical species (e.g. *B. melitensis* 16 M) (Cloeckaert et al., 2001). As a consequence group H and I non-classical *Brucella* spp. (African bullfrog isolates), possess Omp2b porins with an Omp2a-like L8 surface loop at the amino acid sequence level, as seen in all classical *Brucella* species (Supplementary Figure S3). The L8 surface loop was previously shown to be an important target site recognized by monoclonal antibodies directed against conformational epitopes of the Omp2b porin (Paquet et al., 2001). We thus predict that the genetic loss in the L8 encoding region has created antigenic variability of the corresponding protein in the course of evolution of *Brucella* spp., and possibly may have affected antibody induction and recognition in some animal species. In several bacterial species gene conversion appears to have a prime importance in the generation of antigenic variation, an interesting mechanism whereby some bacterial pathogens are able to avoid the host immune system (Santoyo and Romero, 2005). For example, in *Anaplasma marginale* the major surface protein Msp2 is encoded by a single expression site, and diversity is achieved by gene conversion of chromosomally encoded *msp2* pseudogenes (Graça et al., 2015). A model was proposed where *msp2*, through gene conversion, progressively incorporates changes to produce an *msp2* repertoire capable of generating sufficient antigenic variants to escape immunity and establish persistent infection (Graça et al., 2019). We may see a similar situation from the present *omp2* study, with the progressive changes of the Omp2b porin cited above, altering the porin both functionally and antigenically.

## Conclusion

This study provided evidence for a progressive genetic loss in the *omp2b* gene encoding the major outer membrane porin, from non-classical *Brucella* spp. to the classical pathogenic *Brucella* species. This genetic loss appears mainly mediated by segmental gene conversion events with its silent *omp2a* gene copy, and concerns especially the regions encoding the L5 surface loop for porin function and the L8 loop for antigenic variability. The progressive loss in the L5 loop correlates with increasing sugar permeability of the porin, and could be related to environmental adaptation to survive conditions from a possible extracellular aquatic environment (e.g. amphibian or fish) to the intracellular macrophagic environment of the classical pathogenic species causing disease in livestock and humans.

## Data Availability Statement

The new sequence data of this study were deposited in Genbank. The accession numbers are listed in Supplementary Figure S1.

## Author Contributions

AC conceived and designed the study. AC, GV, and MZ analyzed the data, and drafted the manuscript.

## Conflict of Interest

The authors declare that the research was conducted in the absence of any commercial or financial relationships that could be construed as a potential conflict of interest.

## Footnotes

1 www.ebi.ac.uk/Tools/msa/clustalo/
