# Supplementary material for: Omp2b Porin Alteration in the Course of Evolution of Brucella spp
Source: Front Microbiol. 2020 Feb 24;11:284. doi: 10.3389/fmicb.2020.00284 (PMC7050475; doi:10.3389/fmicb.2020.00284)
Supplement: Supplementary file 1 [file Image_1.pdf]

GenBank accession n°	Species	Strain	Host or source	*omp2* locus type	*omp2* gene			90			180			270			360			450			540			630				750			840			930			1020			1080		1140	*omp2* gene	Strain
MN735502	*Brucella* sp.	83-211	Australian rodent	A	*omp2b*																																										*omp2b*	83-211
MN735472					*omp2a*					1	5	1	7	1	11	9	10	5 (14)	8 (19)	7	8	4	6	4	1	3	5 (21)	3 (27)	9	1		1	1	1	2	1	14	2	3	4	4	15 (3)	3 (6)	1	*omp2a*	
MN735503		NF 2627	Australian rodent	A	*omp2b*																																										*omp2b*	NF 2627
MN735473					*omp2a*					1	5	1	7	1	11	9	10	5 (14)	8 (19)	7	8	4	6	4	1	3	5 (21)	3 (27)	9	1		1	1	1	2	1	2	2	3	4	4	15 (3)	3 (6)	1	*omp2a*	
MN735504		NF 2629	Australian rodent	A	*omp2b*																																										*omp2b*	NF 2629
MN735474					*omp2a*					1	5	1	7	1	11	9	10	5 (14)	8 (19)	7	8	4	6	4	1	3	5 (21)	3 (27)	9	1		1	1	1	2	1	14	2	3	4	4	15 (3)	3 (6)	1	*omp2a*	
MN735505		NF 2637	Australian rodent	A	*omp2b*																																										*omp2b*	NF 2637
MN735475					*omp2a*					1	5	1	7	1	11	9	10	5 (14)	8 (19)	7	8	4	6	4	1	3	5 (21)	3 (27)	9	1		1	1	1	2	1	14	2	3	4	4	15 (3)	3 (6)	1	*omp2a*	
MN735506		NF 2640	Australian rodent	A	*omp2b*																																										*omp2b*	NF 2640
MN735476					*omp2a*					1	5	1	7	1	11	9	10	5 (14)	8 (19)	7	8	4	6	4	1	3	5 (21)	3 (27)	9	1		1	1	1	2	1	14	2	3	4	4	15 (3)	3 (6)	1	*omp2a*	
MN735507		NF 2651	Australian rodent	A	*omp2b*																																										*omp2b*	NF 2651
MN735477					*omp2a*					1	5	1	7	1	11	9	10	5 (14)	8 (19)	7	8	4	6	4	1	3	5 (21)	3 (27)	9	1		1	1	1	2	1	14	2	3	4	4	15 (3)	3 (6)	1	*omp2a*	
MN735508		NF 2668	Australian rodent	A	*omp2b*																																										*omp2b*	NF 2668
MN735478					*omp2a*					1	5	1	7	1	11	9	10	5 (14)	8 (19)	7	8	4	6	4	1	3	5 (21)	3 (27)	9	1		1	1	1	2	1	14	2	3	4	4	15 (3)	3 (6)	1	*omp2a*	
MN735509		NF 2810	Australian rodent	A	*omp2b*																																										*omp2b*	NF 2810
MN735479					*omp2a*					1	5	1	7	1	11	9	10	5 (14)	8 (19)	7	8	4	6	4	1	3	5 (21)	3 (27)	9	1		1	1	1	2	1	14	2	3	4	4	15 (3)	3 (6)	1	*omp2a*	
MN735510		NF 2816	Australian rodent	A	*omp2b*																																										*omp2b*	NF 2816
MN735480					*omp2a*					1	5	1	7	1	11	9	10	5 (14)	8 (19)	7	8	4	6	4	1	3	5 (21)	3 (27)	9	1		1	1	1	2	1	14	2	3	4	4	15 (3)	3 (6)	1	*omp2a*	
FM177716.1	*B. inopinata*	BO1	Human	B	*omp2b*						1, **1**	**1**	1, **2**	1	1	1	2, **3**	1			**2**	2, **1**		1, **1**		1	**1**	1, **4**	1	1, **1**						1									*omp2b*	BO1
FM177715.1					*omp2a*					**1**	4, **1**	1, **1**	7	1	11	9	10	5 (12, **2**)	7 (17, **2**)	7	8	4	6	4	**1**	2	5 (21)	3 (27)	9	1	**1**	1	1, **1**	1, **1**	1, **1**	1	14	2, **1**	3	3, **1**	4	15 (3)	3 (6)	1	*omp2a*	
MN735511	*Brucella* sp.	10RB9213	African bullfrog	C	*omp2b*					**1**	1, **1**	**1**	1	1, **1**	1, **1**	**2**		**1**	**1**		**2**	2	**1**			1	1, **3**	1, **2** (21)	1	1, **1**		1	1, **2**			1		1, **1**	2, **3**		1	3			*omp2b*	9213
MN735481					*omp2a*					**1**	4, **1**	1	7	1	11	9	10	5 (12, **2**)	7 (16, **3**)	7	8	4	6	4	1, **1**	2, **2**	5 (21)	3 (27)	9	1	**1**	**2**	1, **1**	1, **1**	1, **1**	1	14	2	3	3	4	15 (3)	3 (6)	1, **1**	*omp2a*	
LT605585.1*	*Brucella* sp.	141012304	Bluespotted ribbontail ray	D	*omp2b*							1	7	1	1		2, **4**	1, **3** (2)	2, **3** (1, **1**)	**2**	1	1, **1**	1	1		1, **1**	1, **9** (5)	1, **1** (28)	4, **3**	1		1	**2**		1	1			2, **2**		1	3			*omp2b*	141012304
LT605585.1*					*omp2a*						4	1	7	1	11	9	10	5 (12, **2**)	7 (19)	7	8	3, **1**	6	4	**2**	2	5 (21)	3 (27)	9	1	**2**	1	1, **1**	1, **1**	2	1	13	2	3	3	4, **1**	15 (3)	3 (6)	1	*omp2a*	
MN735512	*Brucella* sp.	09RB8913	African bullfrog	E	*omp2b*						**1**	1		1, **1**	1		2, **3**	1, **4** (2)	3, **2** (1)	**2**	1, **1**	1, **1**	1	1		1, **1**	1, **9** (5)	1, **1** (28)	4, **3**	1		1	**2**			1			2, **2**		1	3			*omp2b*	8913
MN735482					*omp2a*						4		7	1	11	9	10	5 (12, **2**)	7 (17, **2**)	7	8	4	6	4, **1**	**1**	2, **3**	5 (21)	3 (27)	9, **1**	1	**2**	1	1, **1**	1, **1**	2	1	13	2	3, **1**	3, **1**	4, **1**	15 (3)	3 (6)	1	*omp2a*	
MN735513		09RB8914	African bullfrog	E	*omp2b*						**1**	1		1, **1**	1		2, **3**	1, **4** (2)	3, **2** (1)	**2**	1, **1**	1, **1**	1	1		1, **1**	1, **9** (5)	1, **1** (28)	4, **3**	1		1	**2**			1			2, **2**		1	3			*omp2b*	8914
MN735483					*omp2a*						4		7	1	11	9	10	5 (12, **2**)	7 (17, **2**)	7	8	4	6	4, **1**	**1**	2, **3**	5 (21)	3 (27)	9, **1**	1	**2**	1	1, **1**	1, **1**	2	1	13	2	3, **1**	3, **1**	4, **1**	15 (3)	3 (6)	1	*omp2a*	
MN735514		09RB8915	African bullfrog	E	*omp2b*						**1**	1		1, **1**	1		0	1, **4** (2)	3, **2** (1)	**2**	1, **1**	1, **1**	1	1		1, **1**	1, **9** (5)	1, **1** (28)	4, **3**	1		1	**2**			1			2, **2**		1	3			*omp2b*	8915
MN735484					*omp2a*						4		7	1	11	9	10	5 (12, **2**)	7 (17, **2**)	7	8	4	6	4, **1**	**1**	2, **3**	5 (21)	3 (27)	9, **1**	1	**2**	1	1, **1**	1, **1**	2	1	13	2	3, **1**	3, **1**	4, **1**	15 (3)	3 (6)	1	*omp2a*	
MN735515		09RB8918	African bullfrog	E	*omp2b*						**1**	1		1, **1**	1		2, **3**	1, **4** (2)	3, **2** (1)	**2**	1, **1**	1, **1**	1	1		1, **1**	1, **9** (5)	1, **1** (28)	4, **3**	1		1	**2**			1			2, **2**		1	3			*omp2b*	8918
MN735485					*omp2a*						4		7	1	11	9	10	5 (12, **2**)	7 (17, **2**)	7	8	4	6	4, **1**	**1**	2, **3**	5 (21)	3 (27)	9, **1**	1	**2**	1	1, **1**	1, **1**	2	1	13	2	3, **1**	3, **1**	4, **1**	15 (3)	3 (6)	1	*omp2a*	
MN735516	*Brucella* sp.	09RB8471	African bullfrog	F	*omp2b*						**1**	1		1	1		2, **3**	**4** (2)	2, **3** (1)	**2**	1, **1**	1, **1**	1, **1**	1		1, **1**	1, **9** (5)	1, **1** (28)	4, **3**	1		1	**2**			1			2, **2**		1	3			*omp2b*	8471
MN735486					*omp2a*						4, **1**		7	1	11	9	10	5 (13, **1**)	7 (19)	7	8	4	6	4	**1**	2, **3**	5 (21)	3 (27)	9, **1**	1		1	1, **1**	1, **2**	1, **1**	1	14	2	3	3, **1**	4	15 (3)	3 (6)	1	*omp2a*	
MN735517		10RB9205	African bullfrog	F	*omp2b*						**1**	1		1	1		2, **3**	**4** (2)	2, **3** (1)	**2**	1, **1**	1, **1**	1, **1**	1		1, **1**	1, **9** (5)	1, **1** (28)	4, **3**	1		1	**2**			1			2, **2**		1	3			*omp2b*	9205
MN735487					*omp2a*						4, **1**		7	1	11	9	10	5 (13, **1**)	7 (19)	7	8	4	6	4	**1**	2, **3**	5 (21)	3 (27)	9, **1**	1		1	1, **1**	1, **2**	1, **1**	1	14	2	3	3, **1**	4	15 (3)	3 (6)	1	*omp2a*	
MN735518		10RB9206	African bullfrog	F	*omp2b*						**1**	1		1	1		2, **3**	**4** (2)	2, **3** (1)	**2**	1, **1**	1, **1**	1, **1**	1		1, **1**	1, **9** (5)	1, **1** (28)	4, **3**	1		1	**2**			1			2, **2**		1	3			*omp2b*	9206
MN735488					*omp2a*						4, **1**		7	1	11	9	10	5 (13, **1**)	7 (19)	7	8	4	6	4	**1**	2, **3**	5 (21)	3 (27)	9, **1**	1		1	1, **1**	1, **2**	1, **1**	1	14	2	3	3, **1**	4	15 (3)	3 (6)	1	*omp2a*	
MN735519		10RB9207	African bullfrog	F	*omp2b*						**1**	1		1	1		2, **3**	**4** (2)	2, **3** (1)	**2**	1, **1**	1, **1**	1, **1**	1		1, **1**	1, **9** (5)	1, **1** (28)	4, **3**	1		1	**2**			1			2, **2**		1	3			*omp2b*	9207
MN735489					*omp2a*						4, **1**		7	1	11	9	10	5 (13, **1**)	7 (19)	7	8	4	6	4	**1**	2, **3**	5 (21)	3 (27)	9, **1**	1		1	1, **1**	1, **2**	1, **1**	1	14	2	3	3, **1**	4	15 (3)	3 (6)	1	*omp2a*	
MN735520		10RB9208	African bullfrog	F	*omp2b*						**1**	1		1	1		2, **3**	**4** (2)	2, **3** (1)	**2**	1, **1**	1, **1**	1, **1**	1		1, **1**	1, **9** (5)	1, **1** (28)	4, **3**	1		1	**2**			1			2, **2**		1	3			*omp2b*	9208
MN735490					*omp2a*						4, **1**		7	1	11	9	10	5 (13, **1**)	7 (19)	7	8	4	6	4	**1**	2, **3**	5 (21)	3 (27)	9, **1**	1		1	1, **1**	1, **2**	1, **1**	1	14	2	3	3, **1**	4	15 (3)	3 (6)	1	*omp2a*	
MN735521		10RB9210	African bullfrog	F	*omp2b*						**1**	1		1	1		2, **3**	**4** (2)	2, **3** (1)	**2**	1, **1**	1, **1**	1, **1**	1		1, **1**	1, **9** (5)	1, **1** (28)	4, **3**	1		1	**2**			1			2, **2**		1	3			*omp2b*	9210
MN735491					*omp2a*						4, **1**		7	1	11	9	10	5 (13, **1**)	7 (19)	7	8	4	6	4	**1**	2, **3**	5 (21)	3 (27)	9, **1**	1		1	1, **1**	1, **2**	1, **1**	14	14	2	3	3, **1**	4	15 (3)	3 (6)	1	*omp2a*	
MN735522		10RB9216	African bullfrog	F	*omp2b*						**1**	1		1	1		2, **3**	**4** (2)	2, **3** (1)	**2**	1, **1**	1, **1**	1, **1**	1		1, **1**	1, **9** (5)	1, **1** (28)	4, **3**	1		1	**2**			1			2, **2**		1	3			*omp2b*	9216
MN735492					*omp2a*						4, **1**		7	1	11	9	10	5 (13, **1**)	7 (19)	7	8	4	6	4	**1**	2, **3**	5 (21)	3 (27)	9, **1**	1		1	1, **1**	1, **2**	1, **1**	1	14	2	3	3, **1**	4	15 (3)	3 (6)	1	*omp2a*	
MN735523		10RB9217	African bullfrog	F	*omp2b*						**1**	1		1	1		2, **3**	**4** (2)	2, **3** (1)	**2**	1, **1**	1, **1**	1, **1**	1		1, **1**	1, **9** (5)	1, **1** (28)	4, **3**	1		1	**2**			1			2, **2**		1	3			*omp2b*	9217
MN735493					*omp2a*						4, **1**		7	1	11	9	10	5 (13, **1**)	7 (19)	7	8	4	6	4	**1**	2, **3**	5 (21)	3 (27)	9, **1**	1		1	1, **1**	1, **2**	1, **1**	1	14	2	3	3, **1**	4	15 (3)	3 (6)	1	*omp2a*	
MN735524	*Brucella* sp.	BO2	Human	G	*omp2b*							1, **1**	1	1, **1**	1, **3**	**1**	2, **3**	1, **4** (2)	3, **2** (1)	**2**	1, **2**	2		2, **1**	**1**	1	1, **9** (5)	1, **1** (28)	4, **3**	1		1	**2**		**2**	1			2, **2**		1	3			*omp2b*	BO2
MN735494					*omp2a*						4		7	1	11	9	10	5 (14)	7 (19)	7	8	4	6	4	**1**	2	5 (21)	3 (27)	9	1	**2**	1	1	1	1, **1**	1	14	2	3	3, **1**	4, **1**	15 (3)	3 (6)	1	*omp2a*	
MN735525	*Brucella* sp.	09RB8908	African bullfrog	H	*omp2b*							1, **1**			1		2, **3**	1, **4** (2)	3, **2** (1)	**2**	1	3		2		1	1, **9** (5)	1, **1** (28)	4, **3**	1, **1**	**1**	**2**	**2**		**1**	1				**2**	4	12, **1** (4)	3 (5)		*omp2b*	8908
MN735495					*omp2a*		**1**			1	5	1	7	1	11	9	10	5 (13, **1**)	7 (19)	7	8	4	6	4	**1**	2	5 (21)	3 (27)	9	1	**1**	1	1	1, **1**	1, **1**	1	14	2, **1**	3	3, **1**	4, **1**	15 (3)	3 (6)	1	*omp2a*	
MN735526		09RB8909	African bullfrog	H	*omp2b*							1, **1**			1		2, **3**	1, **4** (2)	3, **2** (1)	**2**	1	3		2		1	1, **9** (5)	1, **1** (28)	4, **3**	1, **1**	**1**	**2**	**2**		**1**	1				**2**	4	12, **1** (4)	3 (5)		*omp2b*	8909
MN735496					*omp2a*		**1**			1	5	1	7	1	11	9	10	5 (13, **1**)	7 (19)	7	8	4	6	4	**1**	2	5 (21)	3 (27)	9	1	**1**	1	1	1, **1**	1, **1**	1	14	2, **1**	3	3, **1**	4, **1**	15 (3)	3 (6)	1	*omp2a*	
MN735527		09RB8910	African bullfrog	H	*omp2b*							1, **1**			1		2, **3**	1, **4** (2)	3, **2** (1)	**2**	1	3		2		1	1, **9** (5)	1, **1** (28)	4, **3**	1, **1**	**1**	**2**	**2**		**1**	1				**2**	4	12, **1** (4)	3 (5)		*omp2b*	8910
MN735497					*omp2a*		**1**			1	5	1	7	1	11	9	10	5 (13, **1**)	7 (19)	7	8	4	6	4	**1**	2	5 (21)	3 (27)	9	1	**1**	1	1	1, **1**	1, **1**	1	14	2, **1**	3	3, **1**	4, **1**	15 (3)	3 (6)	1	*omp2a*	
MN735528		10RB9211	African bullfrog	H	*omp2b*							1, **1**			1		2, **3**	1, **4** (2)	3, **2** (1)	**2**	1	3		2		1	1, **9** (5)	1, **1** (28)	4, **3**	1, **1**	**1**	**2**	**2**		**1**	1				**2**	4	12, **1** (4)	3 (5)		*omp2b*	9211
MN735498					*omp2a*		**1**			1	5	1	7	1	11	9	10	5 (13, **1**)	7 (19)	7	8	4	6	4	**1**	2	5 (21)	3 (27)	9	1	**1**	1	1	1, **1**	1, **1**	1	14	2, **1**	3	3, **1**	4, **1**	15 (3)	3 (6)	1	*omp2a*	
MN735529		10RB9212	African bullfrog	H	*omp2b*							1, **1**			1		2, **3**	1, **4** (2)	3, **2** (1)	**2**	1	3		2		1	1, **9** (5)	1, **1** (28)	4, **3**	1, **1**	**1**	**2**	**2**		**1**	1				**2**	4	12, **1** (4)	3 (5)		*omp2b*	9212
MN735499					*omp2a*		**1**			1	5	1	7	1	11	9	10	5 (13, **1**)	7 (19)	7	8	4	6	4	**1**	2	5 (21)	3 (27)	9	1	**1**	1	1	1, **1**	1, **1**	1	14	2, **1**	3	3, **1**	4, **1**	15 (3)	3 (6)	1	*omp2a*	
MN735530		10RB9214	African bullfrog	H	*omp2b*							1, **1**			1		2, **3**	1, **4** (2)	3, **2** (1)	**2**	1	3		2	**1**	1	1, **9** (5)	1, **1** (28)	4, **3**	1, **1**		**2**	**2**		1, **1**	1				**2**	4	12, **1** (4)	3 (5)		*omp2b*	9214
MN735500					*omp2a*		**1**			1	5	1, **1**	7	1	11	9	10	5 (13, **1**)	7 (19)	7	8	4	6	4	**1**	2	5 (21)	3 (27)	9	1	**1**	1	1	1, **1**	1, **1**	1	14	2, **1**	3	3, **1**	4, **1**	15 (3)	3 (6)	1	*omp2a*	
MN735531	*Brucella* sp.	10RB9215	African bullfrog	I	*omp2b*							1			1		2, **6**	**3** (2)	1, **4** (1)	**2**	1, **1**	3	**1**	2	**1**	1	1, **9** (5)	1, **1** (28)	4, **3**	1, **1**		**2**	1, **1**		**1**	1			**1**	**1**	4	12, **1** (4)	3 (5)		*omp2b*	9215
MN735501					*omp2a*						4		6, **2**	1	11	9	10	5 (12, **2**)	7 (16, **3**)	7	8	4	6	4		2	5 (21)	3 (27)	9, **1**	1		1	1	1, **1**	1, **1**	1	14	2, **1**	3	3, **1**	4	15 (3)	3 (6)	1	*omp2a*	
AM698039	*B. microti*	CCM 4915	Common vole	J	*omp2b*	**1**	**1**					1	**2**		1		2, **6**	1, **2** (2)	1, **4** (1)	**2**	1	3		2	**1**	1	1, **9** (5)	1, **1** (28)	4, **3**	1, **1**		**2**	1, **1**		**1**	1			**1**	**1**	4	12, **1** (4)	3 (5)		*omp2b*	CCM 4915
AM698038					*omp2a*						4		7, **1**	1	11	8, **1**	10	5 (12, **2**)	7 (16, **3**)	7	8	4	6	4		1, **1**	5 (21)	3 (27)	9	1		1	1, **1**	1, **1**	1, **2**	1	13	2	3	4, **2**	4, **1**	15 (3)	3 (6)	1	*omp2a*	
U26440	*B. melitensis*	16M	Goat	K	*omp2b*							1		1	1		2, **6**	1, **2** (2)	1, **4** (1)	**2**	1	3		2	**1**	1	1, **9** (5)	1, **1** (28)	4, **3**	1, **1**		**2**	**1**		**1**	1			**1**	**1**	4	11, **2** (4)	3 (5)		*omp2b*	16M
U26440					*omp2a*		**1**					1	**2**	1	11	8, **1**	10	5 (12, **2**)	7 (16, **3**)	7	8	4	6	4		1, **1**	5 (21)	3 (27)	9	1		1	1, **1**	1, **1**	1, **2**	1	13	2	3	4, **2**	4, **1**	15 (3)	3 (6)	1, **2**	*omp2a*	
U26441	*B. neotomae*	5K33	Desert rat	L	*omp2b*							1	**2**		1		2, **6**	1, **2** (2)	1, **4** (1)	**2**	1	3		2	**1**	1	1, **9** (5)	1, **1** (28)	4, **3**	1, **1**		**2**	**1**		1, **2**	1	13	2	**1**	**1**	4, **2**	12, **1** (4)	3 (5)		*omp2b*	5K33
U26441					*omp2a*			**1**			4		7, **1**	1	11	8, **1**	10	5 (12, **2**)	7 (16, **3**)	7	8	4	6	4, **1**	**1**	1, **1**	5 (21)	3 (27)	9	1		1	1, **1**	1, **1**	1, **2**	1	13	2	**1**	**1**	4, **1**	15 (3)	3 (6)	1	*omp2a*	
AF300818	*B. pinnipedialis*	B2/94	Common seal	M	*omp2b*						4		7, **1**		1		2, **6**	1, **2** (2)	1, **4** (1)	**2**	1	3		2	**1**	1	1, **9** (5)	1, **1** (28)	4, **3**	1, **1**		**2**	1, **1**	1, **1**	1, **2**	1	13	2	3	4, **2**	4	12, **1** (4)	3 (5)		*omp2b*	B2/94
AF300819					*omp2a*						4		7, **1**	1	11	8, **1**	10	5 (12, **2**)	7 (16, **3**)	7	8	4	6	4		1, **1**	5 (21)	3 (27)	9	1		1	1, **1**	1, **1**	1, **2**	1	13	2	3	4, **2**	4, **1**	15 (3)	3 (6)	1	*omp2a*	
AF300816	*B. ceti*	B1/94	Porpoise	N	*omp2b*					**1**	4		7, **1**		1		2, **6**	1, **2** (2)	1, **4** (1)	**2**	1	3		2	**1**	1	1, **9** (5)	1, **1** (28)	4, **3**	1, **1**		1	1, **1**	1, **1**	1, **2**	1	13	2	3	3, **2**	4	12, **1** (4)	3 (5)		*omp2b*	B1/94
AF300817					*omp2a*					**1**	4		7, **1**		1		2, **6**	1, **2** (2)	1, **4** (1)	**2**	1	3		2	**1**	1	1, **9** (5)	1, **1** (28)	4, **3**	1, **1**		1	1, **1**	1, **1**	1, **2**	1	13	2	3	3, **1**	4	14 (4)	4 (5)	1	*omp2a*	
AF300814		B14/94	Common dolphin	O	*omp2b*		**2**					1	**3**		1		2, **6**	1, **2** (2)	1, **4** (1)	**2**	1	3		2	**1**	1	1, **8** (5)	1, **1** (28)	4, **4**	1, **1**		**2**	**1**		**1**	1	13	2	3	4, **2**	4, **1**	14 (4)	4 (5)	1	*omp2b*	B14/94
AF300815					*omp2a*		**2**					1	**3**		1		2, **6**	1, **2** (2)	1, **4** (1)	**2**	1	3		2	**1**	1	1, **8** (5)	1, **1** (28)	4, **4**	1, **1**		**2**	**1**		**1**	1	13	2	3	4, **2**	4, **1**	14 (4)	4 (5)	1	*omp2a*	
KJ510540.1	*B. papionis*	F8/08/60	Baboon	P	*omp2b*							1	**2**		1		2, **6**	1, **2** (2)	1, **4** (1)	**2**	1	3		2	**1**	1	1, **9** (5)	1, **1** (28)	4, **3**	1, **1**		**2**	**1**		**1**	1			**1**	**1**	4, **1**	14 (4)	4 (5)	1	*omp2b*	F8/08/60
KJ493822.1					*omp2a*							1	**2**		1		2, **6**	1, **2** (2)	1, **4** (1)	**2**	1	3		2	**1**	1	1, **9** (5)	1, **1** (28)	4, **3**	1, **1**		**2**	**1**		**1**	1			**1**	**1**	4, **1**	14 (4)	4 (5)	1	*omp2a*	
FR819665.1	*B. vulpis*	F60	Red fox	Q	*omp2b*			**1**			4	1	7	1	11	9	10	5 (13, **1**)	7 (19)	7	8	4	5, **1**	2	**1**	2	5 (21)	3 (27)	7	1		1	1	1, **2**	1, **1**	1	14	2, **1**		**1**	4	13, **1** (3)	3 (6)	1	*omp2b*	F60
FR819663.1					*omp2a*			**1**			4	1	7	1	11	9	10	5 (13, **1**)	7 (19)	7	8	4	5, **1**	2	**1**	2	5 (21)	3 (27)	7	1		1	1	1, **2**	1, **1**	1	14	2, **1**		**1**	4, **2**	15 (3)	3 (6)	1	*omp2a*	

* Sequences taken from the genome sequence assembly (chromosome 1)

Supplementary Figure S1. Schematic multiple nucleotide sequence alignment of the *omp2a* and *omp2b* genes of all *Brucella* strains of this study.
Nucleotide sequences are represented by rectangles divided into boxes of 30 nucleotides. The *Brucella* sp. 83-211 *omp2b* gene sequence was used as a reference (white boxes).
The boxes containing *omp2a*-specific nucleotides are colored in yellow. The numbers in the corresponding boxes indicate the number of *omp2a*-specific nucleotides present in the sequence considered.
Numbers in parentheses represent insertions and deletions. Numbers in red indicate nucleotide differences that are not due to gene conversion.
