# Supplementary material for: Omp2b Porin Alteration in the Course of Evolution of Brucella spp
Source: Front Microbiol. 2020 Feb 24;11:284. doi: 10.3389/fmicb.2020.00284 (PMC7050475; doi:10.3389/fmicb.2020.00284)
Supplement: Supplementary file 2 [file Image_2.pdf]

```
2a_16M             ATGAACATCAAGAGCCTTCTCCTTGGCTCC GCCGCAGCTCTGGTTGCAGCTTCCGGCGCT  60
2a_5K33            ATGAACATCAAGAGCCTTCTCCTTGGCTCC GCTGCAGCTCTGGTTGCAGCTTCCGGCGCT  60
2a_CCM_4915        ATGAACATCAAGAGCCTTCTCCTTGGCTCC GCTGCAGCTCTGGTTGCAGCTTCCGGCGCT  60
2a_B2/94           ATGAACATCAAGAGCCTTCTCCTTGGCTCC GCTGCAGCTCTGGTTGCAGCTTCCGGCGCT  60
2a_10RB9213        ATGAACATCAAGAGCCTTCTCCTTGGCTCC GCTGCAGCTCTGGTTGCAGCTTCCGGCGCT  60
2a_BO1             ATGAACATCAAGAGCCTTCTCCTTGGCTCC GCTGCAGCTCTGGTTGCAGCTTCCGGCGCT  60
2a_10RB9215        ATGAACATCAAGAGCCTTCTCCTTGGCTCC GCTGCAGCTCTGGTTGCAGCTTCCGGCGCT  60
2a_F60             ATGAACATCAAGAGCCTTCTCCTTGGCTCC GCTGCAGCTCTGGTTGCAGCTTCCGGCGCT  60
2b_F60             ATGAACATCAAGAGCCTTCTCCTTGGCTCC GCTGCAGCTCTGGTTGCAGCTTCCGGCGCT  60
2a_141012304       ATGAACATCAAGAGCCTTCTCCTTGGCTCC GCTGCAGCTCTGGTTGCAGCTTCCGGCGCT  60
2a_09RB8913        ATGAACATCAAGAGCCTTCTCCTTGGCTCC GCTGCAGCTCTGGTTGCAGCTTCCGGCGCT  60
2a_83-211          ATGAACATCAAGAGCCTTCTCCTTGGCTCC GCTGCAGCTCTGGTTGCAGCTTCCGGCGCT  60
2a_09RB8471        ATGAACATCAAGAGCCTTCTCCTTGGCTCC GCTGCAGCTCTGGTTGCAGCTTCCGGCGCT  60
2a_BO2             ATGAACATCAAGAGCCTTCTCCTTGGCTCC GCTGCAGCTCTGGTTGCAGCTTCCGGCGCT  60
2a_09RB8908        ATGAACATCAAGAGCCTTCTCCTTGGCTCC GCTGCAGCTCTGGTTGCAGCTTCCGGGGCT  60
2b_10RB9213        ATGAACATCAAGAGCCTTCTCCTTGGCTCC GCTGCAGCTCTGGTTGCAGCTTCCGGCGCT  60
2b_83-211          ATGAACATCAAGAGCCTTCTCCTTGGCTCC GCTGCAGCTCTGGTTGCAGCTTCCGGCGCT  60
2b_BO1             ATGAACATCAAGAGCCTTCTCCTTGGCTCC GCTGCAGCTCTGGTTGCAGCTTCCGGCGCT  60
2b_BO2             ATGAACATCAAGAGCCTTCTCCTTGGCTCC GCTGCAGCTCTGGTTGCAGCTTCCGGCGCT  60
2b_141012304       ATGAACATCAAGAGCCTTCTCCTTGGCTCC GCTGCAGCTCTGGTTGCAGCTTCCGGCGCT  60
2b_09RB8913        ATGAACATCAAGAGCCTTCTCCTTGGCTCC GCTGCAGCTCTGGTTGCAGCTTCCGGCGCT  60
2b_09RB8471        ATGAACATCAAGAGCCTTCTCCTTGGCTCC GCTGCAGCTCTGGTTGCAGCTTCCGGCGCT  60
2b_B2/94           ATGAACATCAAGAGCCTTCTCCTTGGCTCC GCTGCAGCTCTGGTTGCAGCTTCCGGCGCT  60
2a_B1/94           ATGAACATCAAGAGCCTTCTCCTTGGCTCC GCTGCAGCTCTGGTTGCAGCTTCCGGCGCT  60
2b_B1/94           ATGAACATCAAGAGCCTTCTCCTTGGCTCC GCTGCAGCTCTGGTTGCAGCTTCCGGCGCT  60
2a_B14/94          ATGAACATCAAGAGCCTTCTCCTTGGCTCC GCTGCAGTTCTGGTCGCAGCTTCCGGCGCT  60
2b_B14/94          ATGAACATCAAGAGCCTTCTCCTTGGCTCC GCTGCAGTTCTGGTCGCAGCTTCCGGCGCT  60
2b_5K33            ATGAACATCAAGAGCCTTCTCCTTGGCTCC GCTGCAGCTCTGGTTGCAGCTTCCGGCGCT  60
2a_F8/08/60        ATGAACATCAAGAGCCTTCTCCTTGGCTCC GCTGCAGCTCTGGTTGCAGCTTCCGGCGCT  60
2b_F8/08/60        ATGAACATCAAGAGCCTTCTCCTTGGCTCC GCTGCAGCTCTGGTTGCAGCTTCCGGCGCT  60
2b_CCM_4915        ATGAACATCAGGAGCCTTCTCCTTGGCTCC GCTGCAGCTCTGGTTGCGGCTTCCGGCGCT  60
2b_16M             ATGAACATCAAGAGCCTTCTCCTTGGCTCC GCTGCAGCTCTGGTTGCAGCTTCCGGCGCT  60
2b_09RB8908        ATGAACATCAAGAGCCTTCTCCTTGGCTCC GCTGCAGCTCTGGTTGCAGCTTCCGGCGCT  60
2b_10RB9215        ATGAACATCAAGAGCCTTCTCCTTGGCTCC GCTGCAGCTCTGGTTGCAGCTTCCGGCGCT  60
                   ********** ****************** ** **** ****** ** ******** ***

2a_16M             CAGGCTGCCGACGCAATCGTCGCGCCAGAG CCCGAAGCCGTTGAATATGTCCGCGTTTGC  120
2a_5K33            CAGGCTGCCGACGCAATCGTCGCGCCGGAG CCCGAAGCCGTTGAATATGTCCGCGTTTGC  120
2a_CCM_4915        CAGGCTGCCGACGCAATCGTCGCGCCAGAG CCCGAAGCCGTTGAATATGTCCGCGTTTGC  120
2a_B2/94           CAGGCTGCCGACGCAATCGTCGCGCCAGAG CCCGAAGCCGTTGAATATGTCCGCGTTTGC  120
2a_10RB9213        CAGGCTGCCGACGCAATCGTCGCGCCAGAG CCCGAAGCCGTTGAATATGTCCGCGTTTGC  120
2a_BO1             CAGGCTGCCGACGCAATCGTCGCGCCAGAG CCCGAAGCCGTTGAATATGTCCGCGTTTGC  120
2a_10RB9215        CAGGCTGCCGACGCAATCGTCGCGCCAGAG CCCGAAGCCGTTGAATATGTCCGCGTTTGC  120
2a_F60             CAGGCTGCCGACGCAATTGTCGCGCCAGAG CCCGAAGCCGTTGAATATGTCCGCGTTTGC  120
2b_F60             CAGGCTGCCGACGCAATTGTCGCGCCAGAG CCCGAAGCCGTTGAATATGTCCGCGTTTGC  120
2a_141012304       CAGGCTGCCGACGCAATCGTCGCGCCAGAG CCCGAAGCCGTTGAATATGTCCGCGTTTGC  120
2a_09RB8913        CAGGCTGCCGACGCAATCGTCGCGCCAGAG CCCGAAGCCGTTGAATATGTCCGCGTTTGC  120
2a_83-211          CAGGCTGCCGACGCAATCGTCGCGCCAGAG CCCGAAGCCGTTGAATATGTCCGCGTTtGC  120
2a_09RB8471        CAGGCTGCCGACGCAATCGTCGCGCCAGAG CCCGAAGCCGTTGAATATGTCCGCGTTTGC  120
2a_BO2             CAGGCTGCCGACGCAATCGTCGCGCCAGAG CCCGAAGCCGTTGAATATGTCCGCGTTTGC  120
2a_09RB8908        CAGGCTGCCGACGCAATCGTCGCGCCAGAG CCCGAAGCCGTTGAATATGTCCGCGTTTGC  120
2b_10RB9213        CAGGCTGCCGACGCAATCGTCGCGCCAGAG CCCGAAGCCGTTGAATATGTCCGCGTTTGC  120
2b_83-211          CAGGCTGCCGACGCAATCGTCGCGCCAGAG CCCGAAGCCGTTGAATATGTCCGCGTTTGC  120
2b_BO1             CAGGCTGCCGACGCAATCGTCGCGCCAGAG CCCGAAGCCGTTGAATATGTCCGCGTTTGC  120
2b_BO2             CAGGCTGCCGACGCAATCGTCGCGCCAGAG CCCGAAGCCGTTGAATATGTCCGCGTTTGC  120
2b_141012304       CAGGCTGCCGACGCAATCGTCGCGCCAGAG CCCGAAGCCGTTGAATATGTCCGCGTTTGC  120
2b_09RB8913        CAGGCTGCCGACGCAATCGTCGCGCCAGAG CCCGAAGCCGTTGAATATGTCCGCGTTTGC  120
2b_09RB8471        CAGGCTGCCGACGCAATCGTCGCGCCAGAG CCCGAAGCCGTTGAATATGTCCGCGTTTGC  120
2b_B2/94           CAGGCTGCCGACGCAATCGTCGCGCCAGAG CCCGAAGCCGTTGAATATGTCCGCGTTTGC  120
2a_B1/94           CAGGCTGCCGACGCAATCGTCGCGCCAGAG CCCGAAGCCGTTGAATATGTCCGCGTTTGC  120
2b_B1/94           CAGGCTGCCGACGCAATCGTCGCGCCAGAG CCCGAAGCCGTTGAATATGTCCGCGTTTGC  120
2a_B14/94          CAGGCTGCCGACGCAATCGTCGCGCCAGAG CCCGAAGCCGTTGAATATGTCCGCGTTTGC  120
2b_B14/94          CAGGCTGCCGACGCAATCGTCGCGCCAGAG CCCGAAGCCGTTGAATATGTCCGCGTTTGC  120
2b_5K33            CAGGCTGCCGACGCAATCGTCGCGCCAGAG CCCGAAGCCGTTGAATATGTCCGCGTTTGC  120
2a_F8/08/60        CAGGCTGCCGACGCAATCGTCGCGCCAGAG CCCGAAGCCGTTGAATATGTCCGCGTTTGC  120
2b_F8/08/60        CAGGCTGCCGACGCAATCGTCGCGCCAGAG CCCGAAGCCGTTGAATATGTCCGCGTTTGC  120
2b_CCM_4915        CAGGCTGCCGACGCAATCGTCGCGCCAGAG CCCGAAGCCGTTGAATATGTCCGCGTTTGC  120
2b_16M             CAGGCTGCCGACGCAATCGTCGCGCCAGAG CCCGAAGCCGTTGAATATGTCCGCGTTTGC  120
2b_09RB8908        CAGGCTGCCGACGCAATCGTCGCGCCAGAG CCCGAAGCCGTTGAATATGTCCGCGTTTGC  120
2b_10RB9215        CAGGCTGCCGACGCAATCGTCGCGCCAGAG CCCGAAGCCGTTGAATATGTCCGCGTTTGC  120
                   **************** ******** *** ******************************
```

```
                      L1>      <L1
2a_16M           GACGCTTACGGCGCTGGCTACTTCTACATT CCGGGCACCGAAACCTGCCTGCGCGTCCAT  180
2a_5K33          GACGCTTACGGCGCTGGCTACTTCTACATT CCGGGCACCGAAACCTGCCTGCGCATCAGC  180
2a_CCM_4915      GACGCTTACGGCGCTGGCTACTTCTACATT CCGGGCACCGAAACCTGCCTGCGCATCAGC  180
2a_B2/94         GACGCTTACGGCGCTGGCTACTTCTACATT CCGGGCACCGAAACCTGCCTGCGCATCAGC  180
2a_10RB9213      GACGCTTATGGCGCTGGCTACTTCTACATT CCGGGTACCGAAACCTGCCTGCGCATCAGC  180
2a_BO1           GACGCTTACGGCGCTGGTTACTTCTACATT CCGGGCACCGAAACCTGCCTGCGCATTAGC  180
2a_10RB9215      GACGCTTACGGCGCTGGCTACTTCTACATT CCGGGCACCGAAACCTGCCTGCGCATCAGC  180
2a_F60           GACGCTTACGGCGCTGGCTACTTCTACATT CCGGGCACCGAAACCTGCCTGCGCATCAGC  180
2b_F60           GACGCTTACGGCGCTGGCTACTTCTACATT CCGGGCACCGAAACCTGCCTGCGCATCAGC  180
2a_141012304     GACGCTTACGGCGCTGGCTACTTCTACATT CCGGGCACCGAAACCTGCCTGCGCATCAGC  180
2a_09RB8913      GACGCTTACGGCGCTGGCTACTTCTACATT CCGGGCACCGAAACCTGCCTGCGCATCAGC  180
2a_83-211        GACGCTTACGGCGCTGGCTACTTCTATATT CCCGGCACCGAAACCTGCCTGCGCATCAGC  180
2a_09RB8471      GACGCTTACGGCGCTGGCTACTTCTACATT CCGGGCACAGAAACCTGCCTGCGCATCAGC  180
2a_BO2           GACGCTTACGGCGCTGGCTACTTCTACATT CCGGGCACCGAAACCTGCCTGCGCATCAGC  180
2a_09RB8908      GACGCTTACGGCGCTGGCTACTTCTATATT CCCGGCACCGAAACCTGCCTGCGCATCAGC  180
2b_10RB9213      GACGCTTATGGCGCTGGCTACTTCTACATT CCGGGTACCGAAACCTGCCTGCGCATCCAT  180
2b_83-211        GACGCTTACGGCGCTGGCTACTTCTACATT CCGGGCACCGAAACCTGCCTGCGCGTCCAT  180
2b_BO1           GACGCTTACGGCGCTGGCTACTTCTACATT CCGGGTACCGAAACCTGCCTGCGCATCCAT  180
2b_BO2           GACGCTTACGGCGCTGGCTACTTCTACATT CCGGGCACCGAAACCTGCCTGCGCGTCCAT  180
2b_141012304     GACGCTTACGGCGCTGGCTACTTCTACATT CCGGGCACCGAAACCTGCCTGCGCGTCCAT  180
2b_09RB8913      GACGCTTACGGCGCTGGCTACTTCTACATT CCGGGCACAGAAACCTGCCTGCGCGTCCAT  180
2b_09RB8471      GACGCTTACGGCGCTGGCTACTTCTACATT CCGGGCACAGAAACCTGCCTGCGCGTCCAT  180
2b_B2/94         GACGCTTACGGCGCTGGCTACTTCTACATT CCGGGCACCGAAACCTGCCTGCGCATCAGC  180
2a_B1/94         GACGCTTACGGTGCTGGCTACTTCTACATT CCGGGCACCGAAACCTGCCTGCGCATCAGC  180
2b_B1/94         GACGCTTACGGTGCTGGCTACTTCTACATT CCGGGCACCGAAACCTGCCTGCGCATCAGC  180
2a_B14/94        GACGCTTACGGCGCTGGCTACTTCTACATT CCGGGCACCGAAACCTGCCTGCGCGTCCAT  180
2b_B14/94        GACGCTTACGGCGCTGGCTACTTCTACATT CCGGGCACCGAAACCTGCCTGCGCGTCCAT  180
2b_5K33          GACGCTTACGGCGCTGGCTACTTCTACATT CCGGGCACCGAAACCTGCCTGCGCGTCCAT  180
2a_F8/08/60      GACGCTTACGGCGCTGGCTACTTCTACATT CAGGGCACCGAAACCTGCCTGCGCGTCCAT  180
2b_F8/08/60      GACGCTTACGGCGCTGGCTACTTCTACATT CAGGGCACCGAAACCTGCCTGCGCGTCCAT  180
2b_CCM_4915      GACGCTTACGGCGCTGGCTACTTCTACATT CCGGGCACCGAAACCTGCCTGCGCGTCCAT  180
2b_16M           GACGCTTACGGCGCTGGCTACTTCTACATT CCGGGCACCGAAACCTGCCTGCGCGTCCAT  180
2b_09RB8908      GACGCTTACGGCGCTGGCTACTTCTACATT CCGGGCACCGAAACCTGCCTGCGCGTCCAT  180
2b_10RB9215      GACGCTTACGGCGCTGGCTACTTCTACATT CCGGGCACCGAAACCTGCCTGCGCGTCCAT  180
                 ******** ** ***** ******** *** *   ** ** *************** *

                                       L2>
2a_16M           GGTTACGTCCGTTACGACGTAAAGGGCGGC GATGACGTTTACTCCGGTACCGACCGCAAT  240
2a_5K33          GGCTACGTCCGTTACGACGTAAAGGGCGGC GACGACGTTTATACCGGCTCGGATCGTAAA  240
2a_CCM_4915      GGCTACGTCCGTTACGACGTAAAGGGCGGC GACGACGTTTATACCGGCTCGGATCGTAAA  240
2a_B2/94         GGCTACGTCCGTTACGACGTAAAGGGCGGC GACGACGTTTATACCGGCTCGGATCGTAAA  240
2a_10RB9213      GGTTACGTCCGTTACGACGTAAAGGGCGGC GATGACGTTTATACCGGCTCGGATCGTAAA  240
2a_BO1           GGTTACGTCCGTTACGACGTAAAAGGCGGC GATGACGTTTATACCGGCTCGGATCGTAAA  240
2a_10RB9215      GGCTACGTCCGTTACGACGTAAAGGGCGGC GACGACGTCTACACCGGCTCGGATCGTAAA  240
2a_F60           GGTTACGTCCGTTACGACGTAAAGGGCGGC GATGACGTTTATACCGGCTCGGATCGTAAA  240
2b_F60           GGTTACGTCCGTTACGACGTAAAGGGCGGC GATGACGTTTATACCGGCTCGGATCGTAAA  240
2a_141012304     GGTTACGTCCGTTACGACGTAAAGGGCGGC GATGACGTTTATACCGGCTCGGATCGTAAA  240
2a_09RB8913      GGCTACGTCCGTTACGACGTAAAGGGCGGC GATGACGTTTATACCGGCTCGGATCGTAAA  240
2a_83-211        GGTTACGTCCGTTACGACGTAAAGGGCGGC GATGACGTTTATACCGGCTCGGATCGTAAA  240
2a_09RB8471      GGCTACGTCCGTTACGACGTAAAGGGCGGC GATGACGTTTATACCGGCTCGGATCGTAAA  240
2a_BO2           GGCTACGTCCGTTACGACGTAAAGGGCGGC GATGACGTTTATACCGGCTCGGATCGTAAA  240
2a_09RB8908      GGTTACGTCCGTTACGACGTAAAGGGCGGC GATGACGTTTATACCGGCTCGGATCGTAAA  240
2b_10RB9213      GGCTACGTCCGTTACGACGTCAAGGGCGGC GATGACGTTTACTCCGGCACCGACCGTAAG  240
2b_83-211        GGCTACGTCCGTTACGACGTAAAGGGCGGC GATGACGTTTACTCCGGCACCGACCGCAAG  240
2b_BO1           GGCTACGTCCGTTACGACGTGAAGGGCGGC GATAACGTTTACTCCGGTACCGACCGTAAG  240
2b_BO2           GGTTACGTCCGTTACGTCGTAAAGGGCGGC GATGACGTTTACTCCGGCACCGACCGTAAG  240
2b_141012304     GGTTACGTCCGTTACGACGTAAAGGGCGGC GATGACGTTTATACCGGCTCGGATCGTAAA  240
2b_09RB8913      GGTTACGTCCGTTACGACGTAAAGGGCGGC GATGACGTTTACTCCGGCACCGACCGCAAG  240
2b_09RB8471      GGTTACGTCCGTTACGACGTAAAGGGCGGC GATGACGTTTACTCCGGCACCGACCGCAAG  240
2b_B2/94         GGCTACGTCCGTTACGACGTAAAGGGCGGC GACGACGTTTATACCGGCTCGGATCGTAAA  240
2a_B1/94         GGCTACGTCCGTTACGACGTAAAGGGCGGC GACGACGTTTATACCGGCTCGGATCGTAAA  240
2b_B1/94         GGCTACGTCCGTTACGACGTAAAGGGCGGC GACGACGTTTATACCGGCTCGGATCGTAAA  240
2a_B14/94        GGTTACGTCCGTTACGACGTAAAGGGCGGC AATGACGTTTACTCCGGTACCGACCGCAAT  240
2b_B14/94        GGTTACGTCCGTTACGACGTAAAGGGCGGC AATGACGTTTACTCCGGTACCGACCGCAAT  240
2b_5K33          GGTTACGTCCGTTACGACGTAAAGGGCGGC GATGACGTTTACTCCGGTACCGACCGCAAT  240
2a_F8/08/60      GGTTACGTCCGTTACGACGTAAAGGGCGGC GATGACGTTTACTCCGGTACCGACCGCAAT  240
2b_F8/08/60      GGTTACGTCCGTTACGACGTAAAGGGCGGC GATGACGTTTACTCCGGTACCGACCGCAAT  240
2b_CCM_4915      GGTTACGTCCGTTACGACGTAAAGGGCGGC GATGACGTTTACTCCGGTACCGACCGCAAT  240
2b_16M           GGTTACGTCCGTTACGACGTAAAGGGCGGC GATGACGTTTACTCCGGTACCGACCGCAAT  240
2b_09RB8908      GGTTATGTCCGTTACGACGTAAAGGGCGGC GATGACGTTTACTCCGGCACCGACCGCAAG  240
2b_10RB9215      GGTTACGTCCGTTACGACGTAAAGGGCGGC GATGACGTTTACTCCGGCACCGACCGCAAG  240
                 ** ** ********** *** ** ******  *   **** **   ****   * ** ** **
```

```
                                 <L2
2a_16M            GGCTGGGACAAGGGCGCTCGTTTCGCACTC ATGTTCAACACGAATTCGGAAACCGAACTC   300
2a_5K33           GGCTGGGACAAGGGCGCTCGTTTCGCACTC ATGTTCAACACGAATTCGGAAACCGAACTC   300
2a_CCM_4915       GGCTGGGACAAGGGCGCTCGTTTCGCACTC ATGTTCAACACGAATTCGGAAACCGAACTC   300
2a_B2/94          GGCTGGGACAAGGGCGCTCGTTTCGCACTC ATGTTCAACACGAATTCGGAAACCGAACTC   300
2a_10RB9213       GGCTGGGACAAGGGCGCTCGTTTCGCACTC ATGTTCAACACGAATTCGGAAACCGAACTC   300
2a_BO1            GGCTGGGACAAGGGCGCTCGTTTCGCACTC ATGTTCAACACGAATTCGGAAACCGAACTC   300
2a_10RB9215       GGCTGGGACAAGGGCGCTCGTTTCGCACTC ATGTTCAACACGAATTCGGAAACCGAACTC   300
2a_F60            GGCTGGGACAAGGGCGCTCGTTTCGCACTC ATGTTCAACACGAATTCGGAAACCGAACTC   300
2b_F60            GGCTGGGACAAGGGCGCTCGTTTCGCACTC ATGTTCAACACGAATTCGGAAACCGAACTC   300
2a_141012304      GGCTGGGACAAGGGCGCTCGTTTCGCACTC ATGTTCAACACGAATTCGGAAACCGAACTC   300
2a_09RB8913       GGCTGGGACAAGGGCGCTCGTTTCGCACTC ATGTTCAACACGAATTCGGAAACCGAACTC   300
2a_83-211         GGCTGGGACAAGGGCGCTCGTTTCGCACTC ATGTTCAACACGAATTCGGAAACCGAACTC   300
2a_09RB8471       GGCTGGGACAAGGGCGCTCGTTTCGCACTC ATGTTCAACACGAATTCGGAAACCGAACTC   300
2a_BO2            GGCTGGGACAAGGGCGCTCGTTTCGCACTC ATGTTCAACACGAATTCGGAAACCGAACTC   300
2a_09RB8908       GGCTGGGACAAGGGCGCTCGTTTCGCACTC ATGTTCAACACGAATTCGGAAACCGAACTC   300
2b_10RB9213       GGCTGGGACAAGGGTGCTCGTTTCGCACTC CGCCTTTCCACCGGTTCGGAAACCGAACTC   300
2b_83-211         GGCTGGGACAAGAGCGCTCGTTTCGCACTC CGCGTTTCTACCGGTTCGGAAACCGAACTC   300
2b_BO1            GGCTGGGACAAGGGCGCTCGTTTCGCACTC CGCGTTTCCACCGGTTCGGAAACCGAACTC   300
2b_BO2            GGCTGGGACAAGGGTGCTCGTTTCGCACTC CGTCTTTCCACCGGTTCGGAAACCGAGCTC   300
2b_141012304      GGCTGGGACAAGGGCGCTCGTTTCGCACTC CGCGTTTCCACCGGTTCGGAAACCGAACTC   300
2b_09RB8913       GGCTGGGACAAGGGTGCTCGTTTCGCACTC CGCGTTTCCACCGGTTCGGAAACCGAACTC   300
2b_09RB8471       GGCTGGGACAAGGGCGCTCGTTTCGCACTC CGCGTTTCCACCGGTTCGGAAACCGAACTC   300
2b_B2/94          GGCTGGGACAAGAGCGCTCGTTTCGCACTC CGCGTTTCCACCGGTTCGGAAACCGAACTC   300
2a_B1/94          GGCTGGGACAAGAGCGCTCGTTTCGCACTC CGCGTTTCCACCGGTTCGGAAACCGAACTC   300
2b_B1/94          GGCTGGGACAAGAGCGCTCGTTTCGCACTC CGCGTTTCCACCGGTTCGGAAACCGAACTC   300
2a_B14/94         GGCTGGGACAAGAGCGCTCGTTTCGCACTC CGCGTTTCCACCGGTTCGGAAACCGAACTC   300
2b_B14/94         GGCTGGGACAAGAGCGCTCGTTTCGCACTC CGCGTTTCCACCGGTTCGGAAACCGAACTC   300
2b_5K33           GGCTGGGACAAGAGCGCTCGTTTCGCACTC CGCGTTTCCACCGGTTCGGAAACCGAACTC   300
2a_F8/08/60       GGCTGGGACAAGAGCGCTCGTTTCGCACTC CGCGTTTCCACCGGTTCGGAAACCGAACTC   300
2b_F8/08/60       GGCTGGGACAAGAGCGCTCGTTTCGCACTC CGCGTTTCCACCGGTTCGGAAACCGAACTC   300
2b_CCM_4915       GGCTGGGACAAGAGCGCTCGTTTCGCACTC CGCGTTTCCACCGGTTCGGAAACCGAACTC   300
2b_16M            GGCTGGGACAAGGGCGCTCGTTTCGCACTC CGCGTTTCCACCGGTTCGGAAACCGAACTC   300
2b_09RB8908       GGCTGGGACAAGAGCGCTCGTTTCGCACTC CGCGTTTCCACCGGTTCGGAAACCGAACTC   300
2b_10RB9215       GGCTGGGACAAGAGCGCTCGTTTCGCACTC CGCGTTTCCACCGGTTCGGAAACCGAACTC   300
                  ************ * ***************     *    **   ************ ***

                                                                L3>
2a_16M            GGCACACTCGGCACCTATACTCAGCTGCGC TTCAACTACACCAGCAACAATTCACGTCAT   360
2a_5K33           GGCACACTCGGCACCTATACTCAGCTGCGC TTCAACTACACCAGCAACAATTCACGTCAT   360
2a_CCM_4915       GGCACACTCGGCACCTATACTCAGCTGCGC TTCAACTACACCAGCAACAATTCACGTCAT   360
2a_B2/94          GGCACACTCGGCACCTATACTCAGCTGCGC TTCAACTACACCAGCAACAATTCACGTCAT   360
2a_10RB9213       GGCACACTCGGCACCTACACTCAGCTGCGT TTCAACTACACCAGCAACAATTCACGTCAT   360
2a_BO1            GGCACACTCGGCACCTACACTCAGCTGCGT TTCAACTACACCAGCAACAATTCACGTCAT   360
2a_10RB9215       GGCACACTCGGCACCTACACTCAGCTGCGT TTCAACTACACCAGCAACAATTCACGTCAT   360
2a_F60            GGCACACTCGGCACCTACACTCAGCTGCGT TTCAACTACACCAGCAACAATTCACGTCAT   360
2b_F60            GGCACACTCGGCACCTACACTCAGCTGCGT TTCAACTACACCAGCAACAATTCACGTCAT   360
2a_141012304      GGCACACTCGGCACCTACACTCAGCTGCGT TTCAACTACACCAGCAACAATTCACGTCAT   360
2a_09RB8913       GGCACACTCGGCACCTACACTCAGCTGCGT TTCAACTACACCAGCAACAATTCACGTCAT   360
2a_83-211         GGCACACTCGGCACCTACACTCAGCTGCGT TTCAACTACACCAGCAACAATTCACGTCAT   360
2a_09RB8471       GGCACACTCGGCACCTACACTCAGCTGCGT TTCAACTACACCAGCAACAATTCACGTCAT   360
2a_BO2            GGCACACTCGGCACCTACACTCAGCTGCGT TTCAACTACACCAGCAACAATTCACGTCAT   360
2a_09RB8908       GGCACACTCGGCACCTACACTCAGCTGCGT TTCAACTACACCAGCAACAATTCACGTCAT   360
2b_10RB9213       GGCACCTTGAAGACCTTCACCGAACTGCGC TTTAACTACTCTGCAAGCAATTCGCGTGAA   360
2b_83-211         GGCACCCTCAAGACCTTCACCGAACTGCGC TTTAACTACTCTGCAAGCAATTCGCGTGAA   360
2b_BO1            GGCACCCTCAAGACCTACACCGAACTGCGC TTCAACTATGCTGCGAACAATTCGCGTGAA   360
2b_BO2            GGTACCCTCAAGACCTTCACCGAACTGCGC TTCAACTATGCTGCGAACAATTCGCGTGAA   360
2b_141012304      GGCACCCTCAAGACCTTCACCGAACTGCGC TTCAACTATGCTGCGAACAATTCGCGTAAA   360
2b_09RB8913       GGCACCCTCAAGACCTTCACCGAACTGCGC TTCAACTATGCTGCGAACAATTCGCGTGAA   360
2b_09RB8471       GGCACCCTCAAGACCTTCACCGAACTGCGC TTCAACTATGCTGCGAACAATTCGCGTGAA   360
2b_B2/94          GGCACCCTCAAGACCTTCACCGAACTGCGC TTCAACTATGCTGCGAACAATTCGGGCGTA   360
2a_B1/94          GGCACCCTCAAGACCTTCACCGAACTGCGC TTCAACTATGCTGCGAACAATTCGGGCGTA   360
2b_B1/94          GGCACCCTCAAGACCTTCACCGAACTGCGC TTCAACTATGCTGCGAACAATTCGGGCGTA   360
2a_B14/94         GGCACCCTCAAGACCTTCACCGAACTGCGC TTCAACTATGCTGCGAACAATTCGGGCGTA   360
2b_B14/94         GGCACCCTCAAGACCTTCACCGAACTGCGC TTCAACTATGCTGCGAACAATTCGGGCGTA   360
2b_5K33           GGCACCCTCAAGACCTTCACCGAACTGCGC TTCAACTATGCTGCGAACAATTCGGGCGTA   360
2a_F8/08/60       GGCACCCTCAAGACCTTCACCGAACTGCGC TTCAACTATGCTGCGAACAATTCGGGCGTA   360
2b_F8/08/60       GGCACCCTCAAGACCTTCACCGAACTGCGC TTCAACTATGCTGCGAACAATTCGGGCGTA   360
2b_CCM_4915       GGCACCCTCAAGACCTTCACCGAACTGCGC TTCAACTATGCTGCGAACAATTCGGGCGTA   360
2b_16M            GGCACCCTCAAGACCTTCACCGAACTGCGC TTCAACTATGCTGCGAACAATTCGGGCGTA   360
2b_09RB8908       GGCACCCTCAAGACCTTCACCGAACTGCGC TTCAACTATGCTGCGAACAATTCGCGTGAA   360
2b_10RB9215       GGCACCCTCAAGACCTTCACCGAACTGCGC TTCAACTATGCTGCGAACAATTCCGGTGTA   360
                  ** **  *    ****  **  * *****  ** *****  *    * ******  *
```

```
2a_16M            GATGGCCAATACGGCGATTTCAGCGATGAT CGTGATGTCGCTGATGGCGGCGTAAGCACC  420
2a_5K33           GATGGCCAATACGGCGATTTCAGCGATGAT CGTGATGTCGCTGATGGCGGCGTAAGCACC  420
2a_CCM_4915       GATGGCCAATACGGCGATTTCAGCGATGAT CGTGATGTCGCTGATGGCGGCGTAAGCACC  420
2a_B2/94          GATGGCCAATACGGCGATTTCAGCGATGAT CGTGATGTCGCTGATGGCGGCGTAAGCACC  420
2a_10RB9213       GATGGCCAATACGGCGATTTCAGCGATGAT CGTGATGTCGCTGATGGCGGCGTAAGCACC  420
2a_BO1            GATGGCCAATACGGCGATTTCAGCGATGAT CGTGATGTTGCTGATGGCGGCGTAAGCACC  420
2a_10RB9215       GATGGCCAATACGGCGATTTCAGCGATGAT CGTGATGTCGCTGATGGCGGCGTAAGCACC  420
2a_F60            GATGGCCAATACGGCGATTTTAGCGATAGT GTTGATGTTGCTGATGGCGGCGTAAGCACC  420
2b_F60            GATGGCCAATACGGCGATTTTAGCGATAGT GTTGATGTTGCTGATGGCGGCGTAAGCACC  420
2a_141012304      GATGGCCAATACGGCGATTTCAGCGATGAT GTTGATGTTGCTGATGGCGGCGTAAGCACC  420
2a_09RB8913       GATGGCCAATACGGCGATTTCAGCGATGAT CGTGATGTTGCTGATGGCGGCGTAAGCACC  420
2a_83-211         GATGGCCAATACGGCGATTTCAGCGATAGT GTTGATGTTGCTGATGGCGGCGTAAACACC  420
2a_09RB8471       GATGGCCAATACGGCGATTTCAGCGATAAT GTTGATGTTGCTGATGGCGGCGTAAGCACC  420
2a_BO2            GATGGCCAATACGGCGATTTCAGCGATAGT GTTGATGTTGCTGATGGCGGCGTAAGCACC  420
2a_09RB8908       GATGGCCAATACGGCGATTTCAGCGACAGT GTTGATGTTGCTGATGGCGGCGTAAGCACC  420
2b_10RB9213       GATGGTTACTATGGTA-------------- -------------------AGAACAGCGAC  387
2b_83-211         GATGGCTACTATGGTA-------------- -------------------CGAACAGCGAC  387
2b_BO1            GATGGCTACTATGGCA-------------- -------------------CGAACAGCGAC  387
2b_BO2            GATGGTGTTTATGGTGAT------------ ------------------GGCACCAGCAGC  390
2b_141012304      GATGGTTTTTATGGTGAT------------ ------------------GACACCAGCAGC  390
2b_09RB8913       GATGGTGTTTATGGTGAT------------ ------------------GGCACCAGCAGC  390
2b_09RB8471       GATGGTGTTTATGGTAAT------------ ------------------GACACCAGCAGC  390
2b_B2/94          GATGGTAAATATGGTAAT------------ ------------------GAAACCAGCAGC  390
2a_B1/94          GATGGTAAATATGGTAAT------------ ------------------GAAACCAGCAGC  390
2b_B1/94          GATGGTAAATATGGTAAT------------ ------------------GAAACCAGCAGC  390
2a_B14/94         GATGGTAAATATGGTAAT------------ ------------------GAAACCAGCAGC  390
2b_B14/94         GATGGTAAATATGGTAAT------------ ------------------GAAACCAGCAGC  390
2b_5K33           GATGGTAAATATGGTAAT------------ ------------------GAAACCAGCAGC  390
2a_F8/08/60       GATGGTAAATATGGTAAT------------ ------------------GAAACCAGCAGC  390
2b_F8/08/60       GATGGTAAATATGGTAAT------------ ------------------GAAACCAGCAGC  390
2b_CCM_4915       GATGGTAAATATGGTAAT------------ ------------------GAAACCAGCAGC  390
2b_16M            GATGGTAAATATGGTAAT------------ ------------------GAAACCAGCAGC  390
2b_09RB8908       GATGGTGTTTATGGTGAT------------ ------------------GGCACCAGCAGC  390
2b_10RB9215       GATGGTGATTATGGTAAT------------ ------------------GAAACCAGCAGC  390
                  *****    ** **                                       * *  *
                                                   <L3
2a_16M            GGCACCGATCTGCAGTTTGCATATATCACG CTTGGTGGTTTCAAGGTTGGTATCGACGAA  480
2a_5K33           GGCACCGATCTGCAGTTTGCATATATCACG CTTGGTGGTTTCAAGGTTGGTATCGACGAA  480
2a_CCM_4915       GGCACCGATCTGCAGTTTGCATATATCACG CTTGGTGGTTTCAAGGTTGGTATCGACGAA  480
2a_B2/94          GGCACCGATCTGCAGTTTGCATATATCACG CTTGGTGGTTTCAAGGTTGGTATCGACGAA  480
2a_10RB9213       GGCACCGATCTGCAGTTTGCATATATCACG CTTGGTGGTTTCAAGGTTGGTATCGACGAA  480
2a_BO1            GGCACCGATCTGCAGTTTGCATATATCACG CTTGGTGGTTTCAAGGTTGGTATCGACGAA  480
2a_10RB9215       GGCACCGATCTGCAGTTTGCATATATCACG CTTGGTGGTTTCAAGGTTGGTATCGACGAA  480
2a_F60            GGCACCGATCTGCAGTTTGCATATATCACG CTTGGTGGTTTCAAGGTTGGTATCGACGAA  480
2b_F60            GGCACCGATCTGCAGTTTGCATATATCACG CTTGGTGGTTTCAAGGTTGGTATCGACGAA  480
2a_141012304      GGCACCGATCTGCAGTTTGCATATATCACG CTTGGTGGTTTCAAGGTTGGTATCGACGAA  480
2a_09RB8913       GGCACCGATCTGCAGTTTGCATATATCACG CTTGGTGGTTTCAAGGTTGGTATCGACGAA  480
2a_83-211         GGCACCGATCTGCAGTTTGCATATATCACG CTTGGTGGTTTCAAGGTTGGTATCGACGAA  480
2a_09RB8471       GGCACCGATCTGCAGTTTGCATATATCACG CTTGGTGGTTTCAAGGTTGGTATCGACGAA  480
2a_BO2            GGCACCGATCTGCAGTTTGCATATATCACG CTTGGTGGTTTCAAGGTTGGTATCGACGAA  480
2a_09RB8908       GGCACCGATCTGCAGTTTGCATATATCACG CTTGGTGGTTTCAAGGTTGGTATCGACGAA  480
2b_10RB9213       GGCACCGTCATGCAGTTCGCCTATATCCAG CTCGGCGGCCTGCGCGTTGGTATCGATGAG  447
2b_83-211         GGCACCGTCATGCAGTTCGCCTATATCCAG CTCGGTGGTCTGCGCGTTGGTATCGATGAG  447
2b_BO1            GGCACCGTCATGCAGTTCGCCTATATCCAG CTCGGCGGCCTGCGCGTTGGTATCGATGAG  447
2b_BO2            GGCACCGTCATGGAGTTCGCGTATATCCAG CTCGGCGGCCTGCGCGTTGGTATCGATGAA  450
2b_141012304      GGCACCGTCATGGAGTTCGCGTATATCCAG CTCGGTGGTCTGCGCGTTGGTATCGATGAA  450
2b_09RB8913       GGCACCGTCATGGAGTTCGCGTATATCCAG CTCGGTGGCCTGCGCGTTGGTATCGATGAA  450
2b_09RB8471       GGCACCGTCATGGAGTTCGCTTATATCCAG CTCGGTGGCCTGCGCGTTGGTATCGATGAA  450
2b_B2/94          GGCACCGTCATGGAGTTCGCGTATATCCAG CTCGGTGGTCTGCGCGTTGGTATCGATGAA  450
2a_B1/94          GGCACCGTCATGGAGTTCGCGTATATCCAG CTCGGTGGTCTGCGCGTTGGTATCGATGAA  450
2b_B1/94          GGCACCGTCATGAAGTTCGCGTATATCCAG CTCGGTGGTCTGCGCGTTGGTATCGATGAA  450
2a_B14/94         GGCACCGTCATGGAGTTCGCGTATATCCAG CTCGGTGGTCTGCGCGTTGGTATCGATGAA  450
2b_B14/94         GGCACCGTCATGGAGTTCGCGTATATCCAG CTCGGTGGTCTGCGCGTTGGTATCGATGAA  450
2b_5K33           GGCACCGTCATGGAGTTCGCGTATATCCAG CTCGGTGGTCTGCGCGTTGGTATCGATGAA  450
2a_F8/08/60       GGCACCGTCATGGAGTTCGCGTATATCCAG CTCGGTGGTCTGCGCGTTGGTATCGATGAA  450
2b_F8/08/60       GGCACCGTCATGGAGTTCGCGTATATCCAG CTCGGTGGTCTGCGCGTTGGTATCGATGAA  450
2b_CCM_4915       GGCACCGTCATGGAGTTCGCGTATATCCAG CTCGGTGGTCTGCGCGTTGGTATCGATGAA  450
2b_16M            GGCACCGTCATGGAGTTCGCGTATATCCAG CTCGGTGGTCTGCGCGTTGGTATCGATGAA  450
2b_09RB8908       GGCACCGTCATGGAGTTCGCGTATATCCAG CTCGGTGGTCTGCGCGTTGGTATCGATGAA  450
2b_10RB9215       GGCACCGTCATGGAGTTCGCGTATATCCAG CTCGGCGGTCTGCGCGTTGGTATCGATGAA  450
                  *******    ** **** ** ******  * ** ** **  *     *********** **
```

```
                                    L4>
2a_16M          TCCGAATTCCATACCTTCACCGGTTACCTC GGTGATGTCATCAACGATGATGTCGTCGCT  540
2a_5K33         TCCGAATTCCATACCTTCACCGGTTACCTC GGTGATGTCATCAACGATGATGTCGTCGCT  540
2a_CCM_4915     TCCGAATTCCATACCTTCACCGGTTACCTC GGTGATGTCATCAACGATGATGTCGTCGCT  540
2a_B2/94        TCCGAATTCCATACCTTCACCGGTTACCTC GGTGATGTCATCAACGATGATGTCGTCGCT  540
2a_10RB9213     TCCGAATTCCATACCTTCACCGGTTACCTC GGTGATGTCATCAACGATGATGTCGTCGCT  540
2a_BO1          TCCGAATTCCATACCTTCACCGGTTACCTC GGTGATGTCATCAACGATGATGTCGTCGCT  540
2a_10RB9215     TCCGAATTCCATACCTTCACCGGTTACCTC GGTGATGTCATCAACGATGATGTCGTCGCT  540
2a_F60          TCCGAATTCCATACCTTCACCGGTTACCTC GGTGATGTCATCAACGATGATGTTGTCGCT  540
2b_F60          TCCGAATTCCATACCTTCACCGGTTACCTC GGTGATGTCATCAACGATGATGTTGTCGCT  540
2a_141012304    TCCGAATTCCACACCTTCACCGGTTACCTC GGTGATGTCATCAACGATGATGTCGTCGCT  540
2a_09RB8913     TCCGAATTCCATACCTTCACCGGTTACCTC GGTGATGTCATCAACGATGATGTCGTCGCT  540
2a_83-211       TCCGAATTCCATACCTTCACCGGTTACCTC GGTGATGTCATCAACGATGATGTCGTCGCT  540
2a_09RB8471     TCCGAATTCCATACCTTCACCGGTTACCTC GGTGATGTCATCAACGATGATGTCGTCGCT  540
2a_BO2          TCCGAATTCCATACCTTCACCGGTTACCTC GGTGATGTCATCAACGATGATGTCGTCGCT  540
2a_09RB8908     TCCGAATTCCATACCTTCACCGGTTACCTC GGTGATGTCATCAACGATGATGTCGTCGCT  540
2b_10RB9213     TCGGAATTCCAGACCTTCACCGGTTACCTC GGCGATGTCATCAACGATGACGTGATTTCG  507
2b_83-211       TCGGAATTCCAGACCTTCACCGGCTATCTC GGCGATGTCATCAACGATGACGTGATCTCG  507
2b_BO1          TCGGAATTCCAAACCTTCACCGGTTACCTC GGCGATGTCATCAACGATGACGTGATCTCG  507
2b_BO2          TCGGAATTCCATACCTTCACCGGCTACCTC GGCGATGTCATCAACGATGACGTGATCTCG  510
2b_141012304    TCGGAATTCCAAACCTTCACCGGCTACCTC GGCGATGTCATCAACGATGACGTGATCTCT  510
2b_09RB8913     TCGGAATTCCAAACCTTCACCGGCTACCTC GGCGATGTCATCAACGATGACGTGATCTCT  510
2b_09RB8471     TCGGAATTCCAAACCTTCACCGGCTACCTC GGCGATGTCATTAACGATGACGTGATCTCT  510
2b_B2/94        TCGGAATTCCATACCTTCACCGGTTACCTC GGCGATGTCATCAACGATGACGTGATCTCG  510
2a_B1/94        TCGGAATTCCATACCTTCACCGGTTACCTC GGCGATGTCATCAACGATGACGTGATCTCG  510
2b_B1/94        TCGGAATTCCATACCTTCACCGGTTACCTC GGCGATGTCATCAACGATGACGTGATCTCG  510
2a_B14/94       TCGGAATTCCATACCTTCACCGGTTACCTC GGCGATGTCATCAACGATGACGTGATCTCG  510
2b_B14/94       TCGGAATTCCATACCTTCACCGGTTACCTC GGCGATGTCATCAACGATGACGTGATCTCG  510
2b_5K33         TCGGAATTCCATACCTTCACCGGTTACCTC GGCGATGTCATCAACGATGACGTGATCTCG  510
2a_F8/08/60     TCGGAATTCCATACCTTCACCGGTTACCTC GGCGATGTCATCAACGATGACGTGATCTCG  510
2b_F8/08/60     TCGGAATTCCATACCTTCACCGGTTACCTC GGCGATGTCATCAACGATGACGTGATCTCG  510
2b_CCM_4915     TCGGAATTCCATACCTTCACCGGTTACCTC GGCGATGTCATCAACGATGACGTGATCTCG  510
2b_16M          TCGGAATTCCATACCTTCACCGGTTACCTC GGCGATGTCATCAACGATGACGTGATCTCG  510
2b_09RB8908     TCGGAATTCCATACCTTCACCGGTTACCTC GGCGATGTCATCAACGATGACGTGATCTCG  510
2b_10RB9215     TCGGAATTCCATACCTTCACCGGTTACCTC GGCGATGTCATCAACGATGACGTGATCTCC  510
                ** ******** *********** ** *** ** ******** ******** **   *  *
                               <L4
2a_16M          GCTGGCTCCTACCGCACCGGCAAGATCGCC TACACCTTCACCGGCGGAAACGGCTTCTCG  600
2a_5K33         GATGGCTCCTACCGCACCGGCAAGATCGCC TACACCTTCACCGGCGGAAACGGCTTCCCG  600
2a_CCM_4915     GCTGGCTCCTACCGCACCGGCAAGATCGCC TACACCTTCACCGGCGGAAACGGCTTCTCG  600
2a_B2/94        GCTGGCTCCTACCGCACCGGCAAGATCGCC TACACCTTCACCGGCGGAAACGGCTTCTCG  600
2a_10RB9213     GCTGGCTCCTACCGCACCGGCAAGATCGCC TACACCTTCACCGGTGGAAATGGCTTCTCG  600
2a_BO1          GCTGGCTCCTACCGCACCGGCAAGATCGCC TACACCTTCACCGGCGGAAATGGCTTCTCG  600
2a_10RB9215     GCTGGCTCCTACCGCACCGGCAAGATCGCC TACACCTTCACCGGCGGAAACGGCTTCTCG  600
2a_F60          GCTGGCTCCTACCGCACCGGCAAGATCTCG TACACCTTCACTGGCGGAAACGGCTTCTCG  600
2b_F60          GCTGGCTCCTACCGCACCGGCAAGATCTCG TACACCTTCACTGGCGGAAACGGCTTCTCG  600
2a_141012304    GCTGGCTCCTACCGCACCGGCAAGATCGCC TACACCTTTACCGGCGGAAATGGCTTCTCG  600
2a_09RB8913     GCTGGCTCCTACCGCACCGGCAAAATCGCC TACACCTTCACCGGCGGAAATGGCTTCTCG  600
2a_83-211       GCTGGCTCCTACCGCACCGGCAAGATCGCC TACACCTTCACCGGTGGAAACGGCTTCTCG  600
2a_09RB8471     GCTGGCTCCTACCGCACCGGCAAGATCGCC TACACCTTCACCGGCGGAAATGGCTTCTCG  600
2a_BO2          GCTGGCTCCTACCGCACCGGCAAGATCGCC TACACCTTCACCGGCGGAAATGGCTTCTCG  600
2a_09RB8908     GCTGGCTCCTACCGCACCGGCAAGATCGCC TACACCTTTACCGGCGGAAACGGCTTCTCG  600
2b_10RB9213     GCTGGCACCTACCGCACCGGCAAGATTTCG TACACCTTCACCGGCGGAAACGGCTTCTCG  567
2b_83-211       GCTGGCACCTACCGCACCGGCAAGATTTCG TACACCTTCACCGGCGGAAACGGCTTCTCG  567
2b_BO1          GCTGGCACCTACCGCACCGGTAAGATCTCG TACACCTTCACCGGCGGAAACGGCTTCTCG  567
2b_BO2          GCTGGCTCCTACCGCACCGGCAAAATCTCG TACACCTTCACTGGCGGAAACGGCTTCTCG  570
2b_141012304    GCTGGCTCCTACCGCACCGGCAAGATTTCG TACACCTTCACCGGCGGAAACGGCTTCTCG  570
2b_09RB8913     GCTGGCTCCTACCGCACCGGCAAGATTTCG TACACCTTCACCGGCGGAAACGGCTTCTCG  570
2b_09RB8471     GCTGGCTCCTACCGCACCGGCAAGATTTCG TACACCTTCACCGGCGGAAACGGCTTCTCG  570
2b_B2/94        GCTGGCTCCTACCGCACCGGCAAGATCTCG TACACCTTCACTGGCGGAAACGGCTTCTCG  570
2a_B1/94        GCTGGCTCCTACCGCACCGGCAAGATCTCG TACACCTTCACTGGCGGAAACGGCTTCTCG  570
2b_B1/94        GCTGGCTCCTACCGCACCGGCAAGATCTCG TACACCTTCACTGGCGGAAACGGCTTCTCG  570
2a_B14/94       GCTGGCTCCTACCGCACCGGCAAGATCTCG TACACCTTCACTGGCGGAAACGGCTTCTCG  570
2b_B14/94       GCTGGCTCCTACCGCACCGGCAAGATCTCG TACACCTTCACTGGCGGAAACGGCTTCTCG  570
2b_5K33         GCTGGCTCCTACCGCACCGGCAAGATCTCG TACACCTTCACTGGCGGAAACGGCTTCTCG  570
2a_F8/08/60     GCTGGCTCCTACCGCACCGGCAAGATCTCG TACACCTTCACTGGCGGAAACGGCTTCTCG  570
2b_F8/08/60     GCTGGCTCCTACCGCACCGGCAAGATCTCG TACACCTTCACTGGCGGAAACGGCTTCTCG  570
2b_CCM_4915     GCTGGCTCCTACCGCACCGGCAAGATCTCG TACACCTTCACTGGCGGAAACGGCTTCTCG  570
2b_16M          GCTGGCTCCTACCGCACCGGCAAGATCTCG TACACCTTCACTGGCGGAAACGGCTTCTCG  570
2b_09RB8908     GCTGGCTCCTACCGCACCGGCAAGATCTCG TACACCTTCACCGGCGGAAACGGCTTCTCG  570
2b_10RB9215     GCTGGCTCCTACCGCACCGGCAAGATCTCG TACACCTTCACTGGCGGAAACGGCTTCTCG  570
                * **** ************* ** **   *  ******** ** ** ***** ****** **
```

```
                                               L5>
2a_16M              GCTGTGATCGCTCTCGAACAGGGTGGCGAA GACGTTGAC---------------------  639
2a_5K33             GCTGTGATCGCTCTCGAACAGGGTGGCGAA GACGTTGAC---------------------  639
2a_CCM_4915         GCTGTGATCGCTCTCGAACAGGGTGGCGAA GACGTTGAC---------------------  639
2a_B2/94            GCTGTGATCGCTCTCGAACAGGGTGGCGAA GACGTTGAC---------------------  639
2a_10RB9213         GCCGTAATCGCTCTCGAACAGGGTGGCGAG GACGTTGAC---------------------  639
2a_BO1              GCTGTGATCGCTCTCGAACAGGGTGGCGAG GACGTTGAC---------------------  639
2a_10RB9215         GCTGTGATCGCTCTCGAACAGGGTGGCGAG GACGTTGAC---------------------  639
2a_F60              GCTGTGATCGCTCTCGAACAGGGTGGCGAG GACGTTGAC---------------------  639
2b_F60              GCTGTGATCGCTCTCGAACAGGGTGGCGAG GACGTTGAC---------------------  639
2a_141012304        GCTGTGATCGCTCTCGAACAGGGTGGCGAG GACGTTGAC---------------------  639
2a_09RB8913         GCCGTAATCGCTCTCGAACAGGGTGGTGAG GACGTTGAC---------------------  639
2a_83-211           GCTGTGATAGCTCTCGAACAGGGTGGCGAG GACGTTGAC---------------------  639
2a_09RB8471         GCCGTAATCGCTCTCGAACAGGGTGGTGAG GACGTTGAC---------------------  639
2a_BO2              GCTGTGATCGCTCTCGAACAGGGTGGCGAG GACGTTGAC---------------------  639
2a_09RB8908         GCTGTGATCGCTCTCGAACAGGGTGGCGAG GACGTTGAC---------------------  639
2b_10RB9213         GCTGTGATCGCTCTCGAACAGGGTGGCGAC AATGATGGCGGTTACACGCCGACCCTTAAA  627
2b_83-211           GCTGTGATCGCTCTTGAACAGGGTGGCGAC AATGATGGTGGTTACACGCCGGTCTTTAAA  627
2b_BO1              GCTGTGATCGCTCTCGAACAGGGTGGCGAC AATGATGGTGGTTACACGCCGGTCTTTAGA  627
2b_BO2              GCTGTGATCGCTCTCGAACAGGGTGGCGAC AACGACGGTGGTTACACTGGCTCG------  625
2b_141012304        GCTGTGATCGCTCTCGAACAGGGTGGCGAT AACGACGGTGGTTACACTGGCTCG------  625
2b_09RB8913         GCTGTGATCGCTCTCGAACAGGGTGGCGAT AACGACGGTGGTTACACTGGCTCG------  625
2b_09RB8471         GCTGTGATCGCTCTCGAACAGGGTGGCGAT AACGACGGTGGTTACACTGGCTCG------  625
2b_B2/94            GCTGTGATCGCTCTCGAACAGGGTGGCGAC AACGACGGTGGTTACACTGGCACG------  625
2a_B1/94            GCTGTGATCGCTCTCGAACAGGGTGGCGAC AACGACGGTGGTTACACTGGCACG------  625
2b_B1/94            GCTGTGATCGCTCTCGAACAGGGTGGCGAC AACGACGGTGGTTACACTGGCACG------  625
2a_B14/94           GCTGTGATCGCTCTCGAACAGGGTGGCGAC AACGACGGTGGTTACACTGGCGCG------  625
2b_B14/94           GCTGTGATCGCTCTCGAACAGGGTGGCGAC AACGACGGTGGTTACACTGGCGCG------  625
2b_5K33             GCTGTGATCGCTCTCGAACAGGGTGGCGAC AACGACGGTGGTTACACTGGCACG------  625
2a_F8/08/60         GCTGTGATCGCTCTCGAACAGGGTGGCGAC AACGACGGTGGTTACACTGGCACG------  625
2b_F8/08/60         GCTGTGATCGCTCTCGAACAGGGTGGCGAC AACGACGGTGGTTACACTGGCACG------  625
2b_CCM_4915         GCTGTGATCGCTCTCGAACAGGGTGGCGAC AACGACGGTGGTTACACTGGCACG------  625
2b_16M              GCTGTGATCGCTCTCGAACAGGGTGGCGAC AACGACGGTGGTTACACTGGCACG------  625
2b_09RB8908         GCTGTGATCGCTCTCGAACAGGGTGGCGAC AACGACGGTGGTTACACTGGCTCG------  625
2b_10RB9215         GCTGTGATCGCTCTCGAACAGGGTGGCGAC AACGACGGTGGTTACACTGGCACG------  625
                    ** ** ** ***** *********** **    * *   *

                                                     <L5
2a_16M              ---------------------------AAC GATTACACGATCGACGGTTACATGCCGCAC  672
2a_5K33             ---------------------------AAC GATTACACGATCGACGGTTACATGCCGCAC  672
2a_CCM_4915         ---------------------------AAC GATTACACGATCGACGGTTACATGCCGCAC  672
2a_B2/94            ---------------------------AAC GATTACACGATCGACGGTTACATGCCGCAC  672
2a_10RB9213         ---------------------------AAC GATTACACGATCGACGGTTACATGCCGCAC  672
2a_BO1              ---------------------------AAC GATTACACGATCGACGGTTACATGCCGCAC  672
2a_10RB9215         ---------------------------AAC GATTACACAATCGACGGTTACATGCCGCAC  672
2a_F60              ---------------------------AAC GATTACACGATTGACGGCTACATGCCGCAC  672
2b_F60              ---------------------------AAC GATTACACGATTGACGGCTACATGCCGCAC  672
2a_141012304        ---------------------------AAC GATTACACGATCGACGGTTACATGCCGCAC  672
2a_09RB8913         ---------------------------AAC GATTATACGATCGACGGTTACATGCCGCAC  672
2a_83-211           ---------------------------AAC GATTACACGATCGACGGTTACATGCCGCAC  672
2a_09RB8471         ---------------------------AAC GATTATACGATCGACGGTTACATGCCGCAC  672
2a_BO2              ---------------------------AAC GATTACACGATCGACGGTTACATGCCGCAC  672
2a_09RB8908         ---------------------------AAC GATTACACGATCGACGGTTACATGCCGCAC  672
2b_10RB9213         GAGGGC---------------------CAA GGTTACCAGATTGATGGCTACATGCCTGAC  666
2b_83-211           GATAGCCAAGGTCGCGAGATTAATGGCCGA GGTTACCAGATTGATGGCTATATGCCTGAC  687
2b_BO1              GATAGCCAAGGTAACGAGATTAGCGGCCAA GGTTACCAGATTGATGGCTACATGCCTGAC  687
2b_BO2              ---------------------------ACC AACTACCACATCGACGGCTACATGCCTGAC  657
2b_141012304        ---------------------------ACC AACTACCACATCGACGGCTACATGCCTGAC  657
2b_09RB8913         ---------------------------ACC AACTACCACATCGACGGCTACATGCCTGAC  657
2b_09RB8471         ---------------------------ACC AACTACCACATCGACGGCTACATGCCTGAC  657
2b_B2/94            ---------------------------ACC AACTACCACATCGACGGCTACATGCCTGAC  657
2a_B1/94            ---------------------------ACC AACTACCACATCGACGGCTACATGCCTGAC  657
2b_B1/94            ---------------------------ACC AACTACCACATCGACGGCTACATGCCTGAC  657
2a_B14/94           ---------------------------ACC AACTGCCACATCGACGGCTACATGCCTGAC  657
2b_B14/94           ---------------------------ACC AACTGCCACATCGACGGCTACATGCCTGAC  657
2b_5K33             ---------------------------ACC AACTACCACATCGACGGCTACATGCCTGAC  657
2a_F8/08/60         ---------------------------ACC AACTACCACATCGACGGCTACATGCCTGAC  657
2b_F8/08/60         ---------------------------ACC AACTACCACATCGACGGCTACATGCCTGAC  657
2b_CCM_4915         ---------------------------ACC AACTACCACATCGACGGCTACATGCCTGAC  657
2b_16M              ---------------------------ACC AACTACCACATCGACGGCTACATGCCTGAC  657
2b_09RB8908         ---------------------------ACC AACTACCACATCGACGGCTACATGCCTGAC  657
2b_10RB9215         ---------------------------ACC AACTACCACATCGACGGCTACATGCCTGAC  657
                                                      *      ** ** ** ** *****   **
```

```
2a_16M           GTTGTTGGCGGCCTGAAATATGCTGGCGGC TGGGGTTCGATCGCTGGTGTTGTTGCCTAT  732
2a_5K33          GTTGTTGGCGGCCTGAAATATGCTGGCGGC TGGGGTTCGATCGCTGGTGTTGTTGCCTAT  732
2a_CCM_4915      GTTGTTGGCGGCCTGAAATATGCTGGCGGC TGGGGTTCGATCGCTGGTGTTGTTGCCTAT  732
2a_B2/94         GTTGTTGGCGGCCTGAAATATGCTGGCGGC TGGGGTTCGATCGCTGGTGTTGTTGCCTAT  732
2a_10RB9213      GTTGTTGGCGGCCTGAAATATGCTGGCGGC TGGGGTTCGATCGCTGGTGTTGCTGCCTAT  732
2a_BO1           GTTGTTGGCGGCCTGAAATATGCTGGCGGC TGGGGTTCGATCGCTGGTGTTGCTGCCTAT  732
2a_10RB9215      GTTGTTGGCGGCCTGAAATATGCTGGCGGC TGGGGTTCGATCGCTGGTGTTGTTGCCTAT  732
2a_F60           GTTGTTGGCGGCCTGAAATATGCTGGCGGC TGGGGTTCGATCGCTGGTGTTGTTGCCTAT  732
2b_F60           GTTGTTGGCGGCCTGAAATATGCTGGCGGC TGGGGTTCGATCGCTGGTGTTGTTGCCTAT  732
2a_141012304     GTTGTTGGCGGCCTGAAATATGCTGGCGGC TGGGGTTCGATCGCGGGTGTTGCTGCCTAT  732
2a_09RB8913      GTTGTTGGCGGCCTGAAATATGCTGGCGGC TGGGGTTCGATCGCGGGTGTTGCTGCCTAT  732
2a_83-211        GTTGTTGGCGGCCTGAAATATGCTGGCGGC TGGGGTTCGATCGCTGGTGTTGTTGCCTAT  732
2a_09RB8471      GTTGTTGGCGGCCTGAAATATGCTGGCGGC TGGGGTTCGATCGCTGGTGTTGTTGCCTAT  732
2a_BO2           GTTGTTGGCGGCCTGAAATATGCTGGCGGC TGGGGTTCGATCGCTGGTGTTGCGGCCTAT  732
2a_09RB8908      GTTGTTGGCGGCCTGAAATATGCTGGCGGC TGGGGTTCGATCGCTGGTGTTGCTGCCTAT  732
2b_10RB9213      GTTGTTGGTGGCCTGAAATATGCTGGCGGC TGGGGTTCGATCGCTGGTGTTGTTGCCTAT  726
2b_83-211        GTCGTTGGCGGCCTGAAATATGCTGGCGGC TGGGGTTCGATCGCTGGTGTTGTTGCCTAT  747
2b_BO1           GTTGTTGGTGGCCTGAAATATGCTGGCGGC TGGGGTTCGATCGCTGGTGTTGTTGCCTAT  747
2b_BO2           GTTGTTGGCGGCCTGAAATATGCTGGCGGC TGGGGTTCGATCGCTGGTGTTGTTGCCTAT  717
2b_141012304     GTTGTTGGCGGCCTGAAATATGCTGGCGGC TGGGGTTCGATCGCTGGTGTTGTTGCCTAT  717
2b_09RB8913      GTTGTTGGCGGCCTGAAATATGCTGGCGGC TGGGGTTCGATCGCTGGTGTTGTTGCCTAT  717
2b_09RB8471      GTTGTTGGCGGCCTGAAATATGCTGGCGGC TGGGGTTCGATCGCTGGTGTTGTTGCCTAT  717
2b_B2/94         GTTGTTGGCGGCCTGAAGTATGCTGGCGGC TGGGGTTCGATCGCTGGTGTTGTTGCCTAT  717
2a_B1/94         GTTGTTGGCGGCCTGAAGTATGCTGGCGGC TGGGGTTCGATCGCTGGTGTTGTTGCCTAT  717
2b_B1/94         GTTGTTGGCGGCCTGAAGTATGCTGGCGGC TGGGGTTCGATCGCTGGTGTTGTTGCCTAT  717
2a_B14/94        GTTGTTGGCGGCCTGAAGTATGCTGGCGGC TGGGGTTCGATCGCTGGTGTTGTTGCCTAT  717
2b_B14/94        GTTGTTGGCGGCCTGAAGTATGCTGGCGGC TGGGGTTCGATCGCTGGTGTTGTTGCCTAT  717
2b_5K33          GTTGTTGGCGGCCTGAAGTATGCTGGCGGC TGGGGTTCGATCGCTGGTGTTGTTGCCTAT  717
2a_F8/08/60      GTTGTTGGCGGCCTGAAGTATGCTGGCGGC TGGGGTTCGATCGCTGGTGTTGTTGCCTAT  717
2b_F8/08/60      GTTGTTGGCGGCCTGAAGTATGCTGGCGGC TGGGGTTCGATCGCTGGTGTTGTTGCCTAT  717
2b_CCM_4915      GTTGTTGGCGGCCTGAAGTATGCTGGCGGC TGGGGTTCGATCGCTGGTGTTGTTGCCTAT  717
2b_16M           GTTGTTGGCGGCCTGAAGTATGCTGGCGGC TGGGGTTCGATCGCTGGTGTTGTTGCCTAT  717
2b_09RB8908      GTTGTTGGCGGCCTGAAGTATGCTGGCGGC TGGGGTTCGATCGCTGGTGTTGTTGCTTAT  717
2b_10RB9215      GTTGTTGGCGGCCTGAAGTATGCTGGCGGC TGGGGTTCGATCGCTGGTGTTGTTGCCTAT  717
                 ** ***** ******** ************ ************** *******  ** ***
                 L6>                                                     <L6
2a_16M           GACTCGGTCATCGAAGAATGGGCTACAAAG GTTCGTGGCGACGTCAACATCACCGACCGG  792
2a_5K33          GACTCGGTCATCGAAGAATGGGCTACAAAG GTTCGTGGCGACGTCAACATCACCGACCGG  792
2a_CCM_4915      GACTCGGTCATCGAAGAATGGGCTACAAAG GTTCGTGGCGACGTCAACATCACCGACCGG  792
2a_B2/94         GACTCGGTCATCGAAGAATGGGCTACAAAG GTTCGTGGCGACGTCAACATCACCGACCGG  792
2a_10RB9213      GACTCGGTCATCGAAGAATGGGCTGCCAAG GTCCGTGGCGACGTCAATATCACCGACCGG  792
2a_BO1           GACTCGGTCATCGAAGAATGGGCTACAAAG GTCCGTGGCGACGTCAATATCACCGACCGG  792
2a_10RB9215      GACTCGGTCATCGAAGAATGGGCTACAAAG GTCCGTGGCGACGTCAACATCACCGACCGG  792
2a_F60           GACTCGGTCATCGAAGAATGGGCTACAAAG GTCCGTGGCGACGTCAACATCACCGACCGG  792
2b_F60           GACTCGGTCATCGAAGAATGGGCTACAAAG GTCCGTGGCGACGTCAACATCACCGACCGG  792
2a_141012304     GACTCGGTCATCGAAGAATGGGCTACAAAG GTCCGTGGCGACGTCAATATCACCGACCGG  792
2a_09RB8913      GACTCGGTCATCGAAGAATGGGCTACAAAG GTCCGTGGCGACGTCAATATCACCGACCGG  792
2a_83-211        GACTCGGTCATCGAAGAATGGGCTACAAAG GTCCGTGGCGACGTCAACATCACCGACCGG  792
2a_09RB8471      GACTCGGTCATCGAAGAATGGGCTACAAAG GTCCGTGGCGACGTCAATATCACCGACCGG  792
2a_BO2           GACTCGGTCATCGAAGAATGGGCTACAAAG GTCCGTGGCGACGTCAACATCACCGACCGG  792
2a_09RB8908      GACTCGGTCATCGAAGAATGGGCTACAAAG GTCCGTGGCGACGTCAACATCACCGACCGG  792
2b_10RB9213      GACTCGGTCATCGAAGAATGGGCTACAAAG GTCCGCGGCGACGTCAACATCACTGACCGG  786
2b_83-211        GACTCGGTCATCGAAGAATGGGCTACGAAG GTCCGTGGCGACGTCAACATCACCGACCAG  807
2b_BO1           GACTCGGTCATCGAAGAATGGGCTACGAAG GTCCGTGGCGACGTCAACATCACCGACCAG  807
2b_BO2           GACTCGGTCATCGAAGAATGGGCTACAAAG GTTCGCGGCGACGTCAACATCACCGACCAG  777
2b_141012304     GACTCGGTCATCGAAGAATGGGCTACAAAG GTTCGCGGCGACGTCAACATCACCGACCAG  777
2b_09RB8913      GACTCGGTCATCGAAGAATGGGCTACAAAG GTTCGCGGCGACGTCAACATCACCGACCAG  777
2b_09RB8471      GACTCGGTCATCGAAGAATGGGCTACAAAG GTTCGCGGCGACGTCAACATCACCGACCAG  777
2b_B2/94         GACTCGGTCATCGAAGAATGGGCTGCCAAG GTTCGTGGCGACGTCAACATCACCGACCGG  777
2a_B1/94         GACTCGGTCATCGAAGAATGGGCTACAAAG GTTCGTGGCGACGTCAACATCACCGACCGG  777
2b_B1/94         GACTCGGTCATCGAAGAATGGGCTACAAAG GTTCGTGGCGACGTCAACATCACCGACCGG  777
2a_B14/94        GACTCGGTCATCGAAGAATGGGCTGCCAAG GTTCGTGGCGACGTCAACATCACCGACCAG  777
2b_B14/94        GACTCGGTCATCGAAGAATGGGCTGCCAAG GTTCGTGGCGACGTCAACATCACCGACCAG  777
2b_5K33          GACTCGGTCATCGAAGAATGGGCTGCCAAG GTTCGTGGCGACGTCAACATCACCGACCAG  777
2a_F8/08/60      GACTCGGTCATCGAAGAATGGGCTGCCAAG GTTCGTGGCGACGTCAACATCACCGACCAG  777
2b_F8/08/60      GACTCGGTCATCGAAGAATGGGCTGCCAAG GTTCGTGGCGACGTCAACATCACCGACCAG  777
2b_CCM_4915      GACTCGGTCATCGAAGAATGGGCTGCCAAG GTTCGTGGCGACGTCAACATCACCGACCGG  777
2b_16M           GACTCGGTCATCGAAGAATGGGCTGCCAAG GTTCGTGGCGACGTCAACATCACCGACCAG  777
2b_09RB8908      GACTCGGTCATCGAAGAATGGGCTGCCAAG GTTCGCGGCGACGTCAACATCACCGACCAG  777
2b_10RB9215      GACTCGGTCATCGAAGAATGGGCTGCTAAG GTTCGCGGCGACGTCAACATCACCGACCAG  777
                 ************************ * *** ** ** *********** ***** **** *
```

```
2a_16M            TTCTCGGTATGGCTGCAGGGCGCATATTCG TCCGCAGCGACGCCGAACCAGAACTACGGT  852
2a_5K33           TTCTCGGTATGGCTGCAGGGCGCATATTCG TCCGCAGCGACGCCGAACCAGAACTACGGT  852
2a_CCM_4915       TTCTCGGTATGGCTGCAGGGCGCATATTCG TCCGCAGCGACGCCGAACCAGAACTACGGT  852
2a_B2/94          TTCTCGGTATGGCTGCAGGGCGCATATTCG TCCGCAGCGACGCCGAACCAGAACTACGGT  852
2a_10RB9213       TTCTCGGTTTGGCTGCAGGGTGCATATTCG TCCGCAGCGACGCCGAACCAGAACTACGGC  852
2a_BO1            TTCTCGGTTTGGCTGCAGGGTGCATATTCG TCCGCAGCGACGCCGAACCAGAACTACGGC  852
2a_10RB9215       TTCTCGGTTTGGCTGCAGGGTGCATATTCG TCCGCAGCGACGCCGAACCAGAACTACGGC  852
2a_F60            TTCTCGGTATGGCTGCAGGGCGTATATTCG TCCGCGGCGACGCCGAACCAGAACTACGGC  852
2b_F60            TTCTCGGTATGGCTGCAGGGCGTATATTCG TCCGCGGCGACGCCGAACCAGAACTACGGC  852
2a_141012304      TTCTCGGTTTGGCTGCAGGGTGCATATTCG TCTGCTGCTACGCCGAACCAGAACTACGGC  852
2a_09RB8913       TTCTCGGTTTGGCTGCAGGGTGCATATTCG TCTGCTGCTACGCCGAACCAGAACTACGGC  852
2a_83-211         TTCTCGGTTTGGCTGCAGGGCGCATATTCG TCTGCTGCTACGCCGAACCAGAACTACGGC  852
2a_09RB8471       TTCTCGGTATGGCTGCAGGGTGCATATTCG TCCGCAGCGACGCCGAACCAGAACTACGGC  852
2a_BO2            TTCTCGGTTTGGCTGCAGGGCGCATATTCG TCCGCAGCGACGCCGAACCAGAACTACGGC  852
2a_09RB8908       TTCTCGGTTTGGCTGCAGGGTGCATATTCG TCCGCAGCGACGCCGAACCAGAACTACGGC  852
2b_10RB9213       TTCTCGGTTTGGTTGCAGGGCGCATATTCG TCCGCTGCTACGCCGGACCAGAACTACGGC  846
2b_83-211         TTCTCGGTTTGGTTGCAGGGCGCATATTCG TCCGCTGCTACGCCGGACCAGAACTACGGC  867
2b_BO1            TTCTCGGTTTGGTTGCAGGGCGCATATTCG TCCGCTGCTACGCCGGACCAGAACTACGGC  867
2b_BO2            TTCTCGGTTTGGTTGCAGGGCGCATATTCG TCCGATGCTACGCCGGATCAGAACTACGGC  837
2b_141012304      TTCTCGGTTTGGTTGCAGGGCGCATATTCG TCCGCTGCTACGCCGAACCAGAACTACGGC  837
2b_09RB8913       TTCTCGGTTTGGTTGCAGGGCGCATATTCG TCCGCTGCTACGCCGGACCAGAACTACGGC  837
2b_09RB8471       TTCTCGGTTTGGTTGCAGGGCGCATATTCG TCCGCTGCTACGCCGGACCAGAACTACGGC  837
2b_B2/94          TTCTCGGTATGGCTGCAGGGCGCATATTCG TCCGCAGCGACGCCGAACCAGAACTACGGT  837
2a_B1/94          TTCTCGGTATGGCTGCAGGGCGCATATTCG TCCGCAGCGACGCCGAACCAGAACTACGGT  837
2b_B1/94          TTCTCGGTATGGCTGCAGGGCGCATATTCG TCCGCAGCGACGCCGAACCAGAACTACGGT  837
2a_B14/94         TTCTCGGTTTGGTTGCAGGGCGCATATTCG TCCGCTGCTACGCCGGATCAGAACTACGGC  837
2b_B14/94         TTCTCGGTTTGGTTGCAGGGCGCATATTCG TCCGCTGCTACGCCGGATCAGAACTACGGC  837
2b_5K33           TTCTCGGTTTGGTTGCAGGGCGCATATTCG TCCGCAGCGACGCCGAACCAGAACTACGGT  837
2a_F8/08/60       TTCTCGGTTTGGTTGCAGGGCGCATATTCG TCCGCTGCTACGCCGGATCAGAACTACGGC  837
2b_F8/08/60       TTCTCGGTTTGGTTGCAGGGCGCATATTCG TCCGCTGCTACGCCGGATCAGAACTACGGC  837
2b_CCM_4915       TTCTCGGTTTGGTTGCAGGGCGCATATTCG TCCGCTGCTACGCCGGATCAGAACTACGGC  837
2b_16M            TTCTCGGTTTGGTTGCAGGGCGCATATTCG TCCGCTGCTACGCCGGATCAGAACTACGGC  837
2b_09RB8908       TTCTCGGTTTGGTTGCAGGGCGCATATTCG TCCGCTGCTACGCCGGATCAGAACTACGGC  837
2b_10RB9215       TTCTCGGTTTGGTTGCAGGGCGCATATTCG TCTGCTGCTACGCCGGATCAGAACTACGGC  837
                  ******** *** ******* * ******* ** *  ** ****** * ***********
                                                                L7>
2a_16M            CAGTGGGGCGGCGATTGGGCTGTCTGGGGT GGTGCAAAGTTCATTGCCACCGAAAAGGCA  912
2a_5K33           CAGTGGGGCGGCGATTGGGCTGTCTGGGGT GGTGCAAAGTTCATTGCCACCGAAAAGGCA  912
2a_CCM_4915       CAGTGGGGCGGCGATTGGGCTGTCTGGGGT GGTGCAAAGTTCATTGCCACCGAAAAGGCA  912
2a_B2/94          CAGTGGGGCGGCGATTGGGCTGTCTGGGGT GGTGCAAAGTTCATTGCCACCGAAAAGGCA  912
2a_10RB9213       CAGTGGGGCGGCGATTGGGCTGTCTGGGGT GGCGCAAAGTTCATTGCCACCGAAAAGGCA  912
2a_BO1            CAGTGGGGCGGCGATTGGGCTGTCTGGGGT GGCGCAAAGTTCATTGCCACCGAAAAGGCA  912
2a_10RB9215       CAGTGGGGCGGCGATTGGGCTGTCTGGGGT GGCGCAAAGTTCATTGCCACCGAAAAGGCA  912
2a_F60            CAGTGGGGCGGCGATTGGGCTGTCTGGGGT GGCGCAAAGTTCATTGCCACCGAAAAGGCA  912
2b_F60            CAGTGGGGCGGCGATTGGGCTGTCTGGGGT GGCGCAAAGTTCATTGCCACCGAAAAGGCA  912
2a_141012304      CAGTGGGGCGGCGATTGGGCTGTCTGGGGT GGTGCAAAGTTCATTGCCACCGAAAAGGCA  912
2a_09RB8913       CAGTGGGGCGGCGATTGGGCTGTCTGGGGT GGTGCAAAGTTCATTGCCACCGAAAAGGCA  912
2a_83-211         CAGTGGGGCGGCGATTGGGCTGTCTGGGGT GGCGCAAAGTTCATTGCCACCGAAAAGGCA  912
2a_09RB8471       CAGTGGGGCGGCGATTGGGCTGTCTGGGGT GGCGCAAAGTTCATTGCCACCGAAAAGGCA  912
2a_BO2            CAGTGGGGCGGCGATTGGGCTGTCTGGGGT GGCGCAAAGTTCATTGCCACCGAAAAGGCA  912
2a_09RB8908       CAGTGGGGCGGCGATTGGGCTGTCTGGGGT GGCGCAAAGTTCATTGCCACCGAAAAGGCA  912
2b_10RB9213       CAGTGGGGCGGCGATTGGGCTGTCTGGGGT GGTCTGAAGTATCAGGCTACGCAGAAGGCT  906
2b_83-211         CAGTGGGGTGGCGATTGGGCTGTCTGGGGT GGTCTGAAGTATCAGGCTACGCAGAAGGCT  927
2b_BO1            CAGTGGGGCGGCGATTGGGCTGTCTGGGGT GGTCTGAAGTATCAGGCTACGCAGAAGGCT  927
2b_BO2            CAGTGGGGCGGCGATTGGGCTGTCTGGGGT GGTCTGAAGTATCAGGCTACGCAGAAGGCT  897
2b_141012304      CAGTGGGGCGGCGATTGGGCTGTCTGGGGT GGTCTGAAGTATCAGGCTACGCAGAAGGCT  897
2b_09RB8913       CAGTGGGGCGGCGATTGGGCTGTCTGGGGT GGTCTGAAGTATCAGGCTACGCAGAAGGCT  897
2b_09RB8471       CAGTGGGGCGGCGATTGGGCTGTCTGGGGT GGTCTGAAGTATCAGGCTACGCAGAAGGCT  897
2b_B2/94          CAGTGGGGCGGCGATTGGGCTGTCTGGGGT GGTGCAAAGTTCATTGCCACCGAAAAGGCA  897
2a_B1/94          CAGTGGGGCGGCGATTGGGCTGTCTGGGGT GGTGCAAAGTTCATTGCCACCGAAAAGGCA  897
2b_B1/94          CAGTGGGGCGGCGATTGGGCTGTCTGGGGT GGTGCAAAGTTCATTGCCACCGAAAAGGCA  897
2a_B14/94         CAGTGGGGCGGCGATTGGGCTGTCTGGGGT GGTGCAAAGTTCATTGCCACCGAAAAGGCA  897
2b_B14/94         CAGTGGGGCGGCGATTGGGCTGTCTGGGGT GGTGCAAAGTTCATTGCCACCGAAAAGGCA  897
2b_5K33           CAGTGGGGCGGCGATTGGGCTGTCTGGGGT GGTGCAAAGTTCATTGCCACCGAAAAGGCA  897
2a_F8/08/60       CAGTGGGGCGGCGATTGGGCTGTCTGGGGT GGTCTGAAGTATCAGGCTACGCAGAAGGCT  897
2b_F8/08/60       CAGTGGGGCGGCGATTGGGCTGTCTGGGGT GGTCTGAAGTATCAGGCTACGCAGAAGGCT  897
2b_CCM_4915       CAGTGGGGCGGCGATTGGGCTGTCTGGGGT GGTCTGAAGTATCAGGCTACGCAGAAGGCT  897
2b_16M            CAGTGGGGCGGCGATTGGGCTGTCTGGGGT GGTCTGAAGTATCAGGCTACGCAGAAGGCT  897
2b_09RB8908       CAGTGGGGCGGCGATTGGGCTGTCTGGGGT GGTCTGAAGTATCAGGCTACGCAGAAGGCT  897
2b_10RB9215       CAGTGGGGCGGCGATTGGGCTGTCTGGGGT GGTCTGAAGTATCAGGCTACGCAGAAGGCT  897
                  ******** ********************* **    ****     ** **  * *****
```

```
                                                   <L7
2a_16M          ACCTTCAATCTGCAGGCTGCGCATGACGAC TGGGGCAAGACCGCAGTTACCGCCAACGTC  972
2a_5K33         ACCTTCAATCTGCAGGCTGCGCATGACGAC TGGGGCAAGACGGCAGTTACGGCTAACGTT  972
2a_CCM_4915     ACCTTCAATCTGCAGGCTGCGCATGACGAC TGGGGCAAGACCGCAGTTACCGCCAACGTC  972
2a_B2/94        ACCTTCAATCTGCAGGCTGCGCATGACGAC TGGGGCAAGACCGCAGTTACCGCCAACGTC  972
2a_10RB9213     ACCTTCAATCTGCAGGCTGCGCATGACGAC TGGGGCAAGACCGCAGTTACCGCCAACGTC  972
2a_BO1          ACCTTCAATCTGCAGGCTGCGCATGATGAC TGGGGCAAGACCGCAGTTACCGCCAACGTC  972
2a_10RB9215     ACCTTCAATCTGCAGGCTGCGCATGATGAC TGGGGCAAGACCGCAGTTACCGCCAACGTC  972
2a_F60          ACCTTCAATCTGCAGGCTGCGCATGACGAC TGGGGCAAGACCGCAGTTACGGCTAACGTT  972
2b_F60          ACCTTCAATCTGCAGGCTGCCCATGACGAC TGGGGCAAGACCGCAGTTACGGCTAACGTT  972
2a_141012304    ACCTTCAATCTGCAGGCTGCGCATGACGAC TGGGGCAAGACCGCAGTTACCGCCAACGTC  972
2a_09RB8913     ACCTTCAATCTGCAGGCTGCGCATGACGAC TGGGGCAAGACCGCGGTTACCGCCAACGTC  972
2a_83-211       ACCTTCAATCTGCAGGCTGCGCATGACGAC TGGGGCAAGACCGCAGTTACCGCCAACGTC  972
2a_09RB8471     ACCTTCAATCTGCAGGCTGCGCATGACGAC TGGGGCAAGACCGCAGTTACCGCCAACGTC  972
2a_BO2          ACCTTCAATCTGCAGGCTGCGCATGACGAC TGGGGCAAGACCGCAGTTACCGCCAACGTC  972
2a_09RB8908     ACCTTCAATCTGCAGGCTGCGCATGATGAC TGGGGCAAGACCGCAGTTACCGCCAACGTC  972
2b_10RB9213     GCCTTCAATCTGCAGGCTGCGCATGACGAT TGGGGTAAGACCGCCGTCACCGCCAACGTT  966
2b_83-211       GCCTTCAACCTGCAGGCTGCGCATGACGAC TGGGGCAAGACCGCAGTTACGGCTAACGTT  987
2b_BO1          GCCTTCAACCTGCAGGCTGCGCATGACGAC TGGGGCAAGACCGCAGTTACGGCTAACGTT  987
2b_BO2          GCCTTCAACCTGCAGGCTGCGCATGACGAC TGGGGCAAGACCGCCGTCACCGCCAACGTT  957
2b_141012304    GCCTTCAACCTGCAGGCTGCGCATGACGAC TGGGGCAAGACCGCCGTCACCGCCAACGTT  957
2b_09RB8913     GCCTTCAACCTGCAGGCTGCGCATGACGAC TGGGGCAAGACCGCCGTCACCGCCAACGTT  957
2b_09RB8471     GCCTTCAACCTGCAGGCTGCGCATGACGAC TGGGGCAAGACCGCCGTCACCGCCAACGTT  957
2b_B2/94        ACCTTCAATCTGCAGGCTGCGCATGACGAC TGGGGCAAGACCGCAGTTACCGCCAACGTC  957
2a_B1/94        ACCTTCAATCTGCAGGCTGCGCATGACGAC TGGGGCAAGACCGCAGTTACCGCCAACGTC  957
2b_B1/94        ACCTTCAATCTGCAGGCTGCGCATGACGAC TGGGGCAAGACCGCAGTTACCGCCAACGTC  957
2a_B14/94       ACCTTCAATCTGCAGGCTGCGCATGACGAC TGGGGCAAGACCGCAGTTACCGCCAACGTC  957
2b_B14/94       ACCTTCAATCTGCAGGCTGCGCATGACGAC TGGGGCAAGACCGCAGTTACCGCCAACGTC  957
2b_5K33         ACCTTCAATCTGCAGGCTGCGCATGACGAC TGGGGCAAGACGGCAGTTACGGCTAACGTT  957
2a_F8/08/60     GCCTTCAACCTGCAGGCTGCGCATGACGAC TGGGGCAAGACGGCAGTTACGGCTAACGTT  957
2b_F8/08/60     GCCTTCAACCTGCAGGCTGCGCATGACGAC TGGGGCAAGACGGCAGTTACGGCTAACGTT  957
2b_CCM_4915     GCCTTCAACCTGCAGGCTGCGCATGACGAC TGGGGCAAGACGGCAGTTACGGCTAACGTT  957
2b_16M          GCCTTCAACCTGCAGGCTGCGCATGACGAC TGGGGCAAGACGGCAGTTACGGCTAACGTT  957
2b_09RB8908     GCCTTCAACCTGCAGGCTGCGCATGACGAC TGGGGCAAGACCGCAGTTACGGCTAACGTT  957
2b_10RB9215     GCCTTCAACCTGCAGGCTGCGCATGACGAC TGGGGCAAGACCGCAGTTACGGCTAACGTT  957
                 ******* *********** ***** **  ***** ***** ** ** ** ** *****
                                                                       L8>
2a_16M          GCTTATCAGCTCGTTCCCGGATTCACCATT ACGCCGGAAGTTTCCTACACCAAATTTGGT  1032
2a_5K33         GCTTACGAACTGGTTCCTGGCTTCACCGTT ACGCCGGAAGTTTCCTACACCAAATTTGGT  1032
2a_CCM_4915     GCTTATCAGCTCGTTCCCGGATTCACCATT ACGCCGGAAGTTTCCTACACCAAATTTGGT  1032
2a_B2/94        GCTTATCAGCTCGTTCCCGGATTCACCATT ACGCCGGAAGTTTCCTACACCAAATTTGGT  1032
2a_10RB9213     GCTTACCAGCTGGTTCCCGGCTTCACCATT ACGCCGGAAGTTTCCTACACCAAGTTTGGT  1032
2a_BO1          GCTTACCAGCTCGTTCCCGGCTTCACCATT ACGCCGGAAGTTTCCTACACCAAGTTTGGT  1032
2a_10RB9215     GCTTACCAGCTCGTTCCCGGCTTCACCATT ACGCCGGAAGTTTCCTACACCAAGTTTGGT  1032
2a_F60          GCTTACGAACTGGTTCCTGGCTTCACCGTT ACGCCGGAAGTTTCCTACTCCAAATTTGGT  1032
2b_F60          GCTTACGAACTGGTTCCTGGCTTCACCGTT ACGCCGGAAGTTTCCTACACCAAGTTTGGT  1032
2a_141012304    GCTTACCAGCTGGTTCCCGGCTTCACCATT ACGCCGGAAGTTTCCTACACCAAATTTGGT  1032
2a_09RB8913     GCTTACCAGCTCGTTCCCGGCTTCACCATT ACGCCGGAAGTTTCCTACACCAAATTTGGT  1032
2a_83-211       GCTTATCAGCTGGTTCCCGGCTTCACCATT ACGCCGGAAGTTTCCTACACCAAGTTTGGT  1032
2a_09RB8471     GCTTACCAGCTCGTTCCCGGCTTCACCATT ACGCCGGAAGTTTCCTACACCAAGTTTGGT  1032
2a_BO2          GCTTACCAGCTCGTTCCCGGCTTCACCATT ACGCCGGAAGTTTCCTACACCAAATTTGGT  1032
2a_09RB8908     GCTTACCAGCTCGTTCCCGGCTTCACCATT ACGCCGGAAGTTTCCTACACCAAATTTGGT  1032
2b_10RB9213     GCTTACGAACTGGTTCCTGGCTTCACCATT ACGCCGGAAGTTTCCTACACCAAGTTCAGC  1026
2b_83-211       GCTTACGAACTGGTTCCTGGCTTCACCATT ACGCCGGAAGTGTCCTACACCAAGTTCAGC  1047
2b_BO1          GCTTACGAACTGGTTCCTGGCTTCACCATT ACGCCGGAAGTGTCCTACACCAAGTTCAGC  1047
2b_BO2          GCTTACGAACTGGTTCCTGGCTTCACCATT ACGCCGGAAGTTTCCTACACCAAGTTCAGC  1017
2b_141012304    GCTTACGAACTGGTTCCTGGCTTCACCATT ACGCCGGAAGTTTCCTACACCAAGTTCAGC  1017
2b_09RB8913     GCTTACGAACTGGTTCCTGGCTTCACCATT ACGCCGGAAGTTTCCTACACCAAGTTCAGC  1017
2b_09RB8471     GCTTACGAACTGGTTCCTGGCTTCACCATT ACGCCGGAAGTTTCCTACACCAAGTTCAGC  1017
2b_B2/94        GCTTATCAGCTCGTTCCCGGCTTCACCGTT ACGCCGGAAGTTTCCTACACCAAGTTTGGT  1017
2a_B1/94        GCTTATCAGCTCGTTCCTGGCTTCACCGTT ACGCCGGAAGTTTCCTACACCAAGTTTGGT  1017
2b_B1/94        GCTTATCAGCTCGTTCCTGGCTTCACCGTT ACGCCGGAAGTTTCCTACACCAAGTTTGGT  1017
2a_B14/94       GCTTATCAGCTCGTTCCCGGATTCACCATT ACGCCGGAAGTTTCCTACACCAAATTTGGT  1017
2b_B14/94       GCTTATCAGCTCGTTCCCGGATTCACCATT ACGCCGGAAGTTTCCTACACCAAATTTGGT  1017
2b_5K33         GCTTACGAACTGGTTCCTGGCTTCACCGTT GCTCCGGAAGTTTCCTACACCAAGTTTGGT  1017
2a_F8/08/60     GCTTACGAACTGGTTCCTGGCTTCACCGTT ACGCCGGAAGTTTCCTACACCAAATTTGGT  1017
2b_F8/08/60     GCTTACGAACTGGTTCCTGGCTTCACCGTT ACGCCGGAAGTTTCCTACACCAAATTTGGT  1017
2b_CCM_4915     GCTTACGAACTGGTTCCTGGCTTCACCGTT ACGCCGGAAGTTTCCTACACCAAGTTTGGT  1017
2b_16M          GCTTACGAACTGGTTCCTGGCTTCACCGTT ACGCCGGAAGTTTCCTACACCAAGTTTGGT  1017
2b_09RB8908     GCTTACGAACTGGTTCCTGGCTTCACTGTT ACGCCGGAAGTTTCCTACACCAAGTTTGGT  1017
2b_10RB9215     GCTTACGAACTGGTTCCTGGCTTCACTGTT ACGCCGGAAGTTTCCTACACCAAGTTTGGT  1017
                *****  * ** ***** ** *****  **  * ******** ****** **** **  *
```

```
                                                                   <L8
2a_16M           GGCGAGTGGAAAGACACCGTTGCTGA---- -----AGACAATGCCTGGGGCGGTATCGTT  1083
2a_5K33          GGCGAGTGGAAAGACACCGTTGCTGA---- -----AGACAATGCCTGGGGCGGTATCGTT  1083
2a_CCM_4915      GGCGAGTGGAAAGACACCGTTGCTGA---- -----AGACAATGCCTGGGGCGGTATCGTT  1083
2a_B2/94         GGCGAGTGGAAAGACACCGTTGCTGA---- -----AGACAATGCCTGGGGCGGTATCGTT  1083
2a_10RB9213      GGCGAGTGGAAAGACACCGTTGCTGA---- -----AGACAATGCCTGGGGCGGTATCGTT  1083
2a_BO1           GGCGAGTGGAAAGACACCGTTGCTGA---- -----AGACAATGCCTGGGGCGGTATCGTT  1083
2a_10RB9215      GGCGAGTGGAAAGACACCGTTGCTGA---- -----AGACAATGCCTGGGGCGGTATCGTT  1083
2a_F60           GGCGAGTGGAAAGACACCGTTGCTGA---- -----AGACAATGCCTGGGGCGGTATCGTT  1083
2b_F60           GGCGAGTGGAAGAACACCGTTGCTGA---- -----AGACAATGCCTGGGGCGGTATCGTT  1083
2a_141012304     GGCGAGTGGAAAGACACCGTTGCTGA---- -----AGACAATGCCTGGGGCGGTATCGTT  1083
2a_09RB8913      GGCGAGTGGAAAGACACCGTTGCTGA---- -----AGACAATGCCTGGGGCGGTATCGTT  1083
2a_83-211        GGCGAGTGGAAAGACACCGTTGCTGA---- -----AGACAATGCCTGGGGCGGTATCGTT  1083
2a_09RB8471      GGCGAGTGGAAAGACACCGTTGCTGA---- -----AGACAATGCCTGGGGCGGTATCGTT  1083
2a_BO2           GGCGAGTGGAAAGACACCGTTGCTGA---- -----AGACAATGCCTGGGGCGGTATCGTT  1083
2a_09RB8908      GGCGAGTGGAAAGACACCGTTGCTGA---- -----AGACAATGCCTGGGGCGGTATCGTT  1083
2b_10RB9213      AACGAGTGGAAGCGTGAGCTTGCTAACGAT ACCCTCGATGATGCTTGGGGCGGTATCGTT  1086
2b_83-211        AACGAGTGGAAGCGTGAGCTCGGCAACGAT ACCCTCGATGATGCTTGGGGCGGTATCGTT  1107
2b_BO1           AACGAGTGGAAGCGTGAGCTCGGCAACGAT ACCCTCGATGATGCTTGGGGCGGTATCGTT  1107
2b_BO2           AACGAGTGGAAGCGTCAGCTTGCTAACGAT ACCCTCGATGATGCTTGGGGCGGTATCGTT  1077
2b_141012304     AACGAGTGGAAGCGTCAGCTTGCTAACGAT ACCCTCGATGATGCTTGGGGCGGTATCGTT  1077
2b_09RB8913      AACGAGTGGAAGCGTCAGCTTGCTAACGAT ACCCTCGATGATGCTTGGGGCGGTATCGTT  1077
2b_09RB8471      AACGAGTGGAAGCGTCAGCTTGCTAACGAT ACCCTCGATGATGCTTGGGGCGGTATCGTT  1077
2b_B2/94         GGCGAGTGGAAGAACACCGTTGCTGA---- -----AGACAATGCTTGGGGCGGTATCGTT  1068
2a_B1/94         GGCGAGTGGAAAGACACCGTTGCTGA---- -----AGACAATGCCTGGGGCGGTATCGTT  1068
2b_B1/94         GGCGAGTGGAAGAACACCGTTGCTGA---- -----AGACAATGCTTGGGGCGGTATCGTT  1068
2a_B14/94        GGCGAGTGGAAAGACACCGTTGCTGA---- -----AGACAATGCCTGGGGCGGTATCGTT  1068
2b_B14/94        GGCGAGTGGAAAGACACCGTTGCTGA---- -----AGACAATGCCTGGGGCGGTATCGTT  1068
2b_5K33          GGCGAGTGGAAGAACACCGTTGCTGA---- -----AGACAATGCTTGGGGCGGTATCGTT  1068
2a_F8/08/60      GGCGAGTGGAAAGACACCGTTGCTGA---- -----AGACAATGCCTGGGGCGGTATCGTT  1068
2b_F8/08/60      GGCGAGTGGAAAGACACCGTTGCTGA---- -----AGACAATGCCTGGGGCGGTATCGTT  1068
2b_CCM_4915      GGCGAGTGGAAGAACACCGTTGCTGA---- -----AGACAATGCTTGGGGCGGTATCGTT  1068
2b_16M           GGCGAGTGGAAGAACACTGTTGCTGA---- -----AGACAATGCTTGGGGCGGTATCGTT  1068
2b_09RB8908      GGCGAGTGGAAGAACACCGTTGCTGA---- -----AGACAATGCTTGGGGCGGTATCGTT  1068
2b_10RB9215      GGCGAGTGGAAGAACACCGTTGCTGA---- -----AGACAATGCTTGGGGCGGTATCGTT  1068
                   *********        * *   *           **  **** ***************

2a_16M           CCTTTCCAGCGCTCGTTCTAA     1104
2a_5K33          CGCTTCCAGCGCTCGTTCTAA     1104
2a_CCM_4915      CGCTTCCAGCGCTCGTTCTAA     1104
2a_B2/94         CGCTTCCAGCGCTCGTTCTAA     1104
2a_10RB9213      CGCTTCCAGCGCTCCTTCTAA     1104
2a_BO1           CGCTTCCAGCGCTCGTTCTAA     1104
2a_10RB9215      CGCTTCCAGCGCTCGTTCTAA     1104
2a_F60           CGCTTCCAGCGCTCGTTCTAA     1104
2b_F60           CGCTTCCAGCGCTCGTTCTAA     1104
2a_141012304     CGCTTCCAGCGCTCGTTCTAA     1104
2a_09RB8913      CGCTTCCAGCGCTCGTTCTAA     1104
2a_83-211        CGCTTCCAGCGCTCGTTCTAA     1104
2a_09RB8471      CGCTTCCAGCGCTCGTTCTAA     1104
2a_BO2           CGCTTCCAGCGCTCGTTCTAA     1104
2a_09RB8908      CGCTTCCAGCGCTCGTTCTAA     1104
2b_10RB9213      CGCTTCCAGCGCTCGTTCTAA     1107
2b_83-211        CGCTTCCAGCGTTCGTTCTAA     1128
2b_BO1           CGCTTCCAGCGTTCGTTCTAA     1128
2b_BO2           CGCTTCCAGCGTTCGTTCTAA     1098
2b_141012304     CGCTTCCAGCGTTCGTTCTAA     1098
2b_09RB8913      CGCTTCCAGCGTTCGTTCTAA     1098
2b_09RB8471      CGCTTCCAGCGTTCGTTCTAA     1098
2b_B2/94         CGCTTCCAGCGTTCGTTCTAA     1089
2a_B1/94         CGCTTCCAGCGCTCGTTCTAA     1089
2b_B1/94         CGCTTCCAGCGTTCGTTCTAA     1089
2a_B14/94        CGCTTCCAGCGCTCGTTCTAA     1089
2b_B14/94        CGCTTCCAGCGCTCGTTCTAA     1089
2b_5K33          CGCTTCCAGCGTTCGTTCTAA     1089
2a_F8/08/60      CGCTTCCAGCGCTCGTTCTAA     1089
2b_F8/08/60      CGCTTCCAGCGCTCGTTCTAA     1089
2b_CCM_4915      CGCTTCCAGCGTTCGTTCTAA     1089
2b_16M           CGCTTCCAGCGTTCGTTCTAA     1089
2b_09RB8908      CGCTTCCAGCGTTCGTTCTAA     1089
2b_10RB9215      CGCTTCCAGCGTTCGTTCTAA     1089
                 *  ******** ** ******
```

Supplementary Figure S2. Multiple nucleotide sequence alignment of *omp2a* and *omp2b* genes, from representative *Brucella* strains containing insertions or deletions. Strains and *omp2* genes used are indicated on the left, *omp2a* genes are indicated as 2a highlighted in yellow. *omp2a*-specific nucleotides are highlighted in yellow, according to the *omp2a* and

omp2b reference sequences used from *Brucella* sp. 83-211. Nucleotides colored in red indicate differences that are not due to gene conversion, according to the same reference sequences. The regions encoding surface loops are indicated above each alignment segment by the letter L and corresponding loop number highlighted in blue, with > and < symbols for the start and end of the loop respectively.
