# Supplementary material for: Omp2b Porin Alteration in the Course of Evolution of Brucella spp
Source: Front Microbiol. 2020 Feb 24;11:284. doi: 10.3389/fmicb.2020.00284 (PMC7050475; doi:10.3389/fmicb.2020.00284)
Supplement: Supplementary file 3 [file Image_3.pdf]

```
                                   B1     L1    B2              B3                L2
83-211         ADAIVAPEPEAVEYVRVCDAYGAGYFYIPGTETCLRVHGYVRYDVKGGDDVYSGTDRKGW   60
BO1            ADAIVAPEPEAVEYVRVCDAYGAGYFYIPGTETCLRIHGYVRYDVKGGDNVYSGTDRKGW   60
10RB9213       ADAIVAPEPEAVEYVRVCDAYGAGYFYIPGTETCLRIHGYVRYDVKGGDDVYSGTDRKGW   60
141012304      ADAIVAPEPEAVEYVRVCDAYGAGYFYIPGTETCLRVHGYVRYDVKGGDDVYTGSDRKGW   60
09RB8913       ADAIVAPEPEAVEYVRVCDAYGAGYFYIPGTETCLRVHGYVRYDVKGGDDVYSGTDRKGW   60
09RB8471       ADAIVAPEPEAVEYVRVCDAYGAGYFYIPGTETCLRVHGYVRYDVKGGDDVYSGTDRKGW   60
BO2            ADAIVAPEPEAVEYVRVCDAYGAGYFYIPGTETCLRVHGYVRYVVKGGDDVYSGTDRKGW   60
09RB8908       ADAIVAPEPEAVEYVRVCDAYGAGYFYIPGTETCLRVHGYVRYDVKGGDDVYSGTDRKGW   60
10RB9215       ADAIVAPEPEAVEYVRVCDAYGAGYFYIPGTETCLRVHGYVRYDVKGGDDVYSGTDRKGW   60
CCM 4915       ADAIVAPEPEAVEYVRVCDAYGAGYFYIPGTETCLRVHGYVRYDVKGGDDVYSGTDRNGW   60
16M            ADAIVAPEPEAVEYVRVCDAYGAGYFYIPGTETCLRVHGYVRYDVKGGDDVYSGTDRNGW   60
B14/94         ADAIVAPEPEAVEYVRVCDAYGAGYFYIPGTETCLRVHGYVRYDVKGGNDVYSGTDRNGW   60
B14/94         ADAIVAPEPEAVEYVRVCDAYGAGYFYIPGTETCLRVHGYVRYDVKGGNDVYSGTDRNGW   60
F8/08/60       ADAIVAPEPEAVEYVRVCDAYGAGYFYIQGTETCLRVHGYVRYDVKGGDDVYSGTDRNGW   60
F8/08/60       ADAIVAPEPEAVEYVRVCDAYGAGYFYIQGTETCLRVHGYVRYDVKGGDDVYSGTDRNGW   60
76-250         ADAIVAPEPEAVEYVRVCDAYGAGYFYIPGTETCLRISGYVRYDVKGGDDVYTGSDRKGW   60
45/20          ADAIVAPEPEAVEYVRVCDAYGAGYFYIPGTETCLRVHGYVRYDVKGGDDVYSGTDRNGW   60
F60            ADAIVAPEPEAVEYVRVCDAYGAGYFYIPGTETCLRISGYVRYDVKGGDDVYTGSDRKGW   60
F60            ADAIVAPEPEAVEYVRVCDAYGAGYFYIPGTETCLRISGYVRYDVKGGDDVYTGSDRKGW   60
16M            ADAIVAPEPEAVEYVRVCDAYGAGYFYIPGTETCLRVHGYVRYDVKGGDDVYSGTDRNGW   60
CCM 4915       ADAIVAPEPEAVEYVRVCDAYGAGYFYIPGTETCLRISGYVRYDVKGGDDVYTGSDRKGW   60
83-211         ADAIVAPEPEAVEYVRVCDAYGAGYFYIPGTETCLRISGYVRYDVKGGDDVYTGSDRKGW   60
               **************************** *******: ***** ****::**:*:**:**
                L2   B4                   B5                  L3
83-211         DKSARFALRVSTGSETELGTLKTFTELRFNYSASNSREDGYYGTN-----------SDGT   109
BO1            DKGARFALRVSTGSETELGTLKTYTELRFNYAANNSREDGYYGTN-----------SDGT   109
10RB9213       DKGARFALRLSTGSETELGTLKTFTELRFNYSASNSREDGYYGKN-----------SDGT   109
141012304      DKGARFALRVSTGSETELGTLKTFTELRFNYAANNSRKDGFYGDDT----------SSGT   110
09RB8913       DKGARFALRVSTGSETELGTLKTFTELRFNYAANNSREDGVYGDGT----------SSGT   110
09RB8471       DKGARFALRVSTGSETELGTLKTFTELRFNYAANNSREDGVYGNDT----------SSGT   110
BO2            DKGARFALRLSTGSETELGTLKTFTELRFNYAANNSREDGVYGDGT----------SSGT   110
09RB8908       DKSARFALRVSTGSETELGTLKTFTELRFNYAANNSREDGVYGDGT----------SSGT   110
10RB9215       DKSARFALRVSTGSETELGTLKTFTELRFNYAANNSGVDGDYGNET----------SSGT   110
CCM 4915       DKSARFALRVSTGSETELGTLKTFTELRFNYAANNSGVDGKYGNET----------SSGT   110
16M            DKGARFALRVSTGSETELGTLKTFTELRFNYAANNSGVDGKYGNET----------SSGT   110
B14/94         DKSARFALRVSTGSETELGTLKTFTELRFNYAANNSGVDGKYGNET----------SSGT   110
B14/94         DKSARFALRVSTGSETELGTLKTFTELRFNYAANNSGVDGKYGNET----------SSGT   110
F8/08/60       DKSARFALRVSTGSETELGTLKTFTELRFNYAANNSGVDGKYGNET----------SSGT   110
F8/08/60       DKSARFALRVSTGSETELGTLKTFTELRFNYAANNSGVDGKYGNET----------SSGT   110
76-250         DKGARFALMFNTNSETELGTLGTYTTLRFNYTSNNSRHDGQYGDFSDDRDVADGSVSTGT   120
45/20          DKSARFALRVSTGSETELGTLKTFTELRFNYAANNSGVDGKYGNET----------SSGT   110
F60            DKGARFALMFNTNSETELGTLGTYTQLRFNYTSNNSRHDGQYGDFSDSVDVADGGVSTGT   120
F60            DKGARFALMFNTNSETELGTLGTYTQLRFNYTSNNSRHDGQYGDFSDSVDVADGGVSTGT   120
16M            DKGARFALMFNTNSETELGTLGTYTQLRFNYTSNNSRHDGQYGDFSDDRDVADGGVSTGT   120
CCM 4915       DKGARFALMFNTNSETELGTLGTYTQLRFNYTSNNSRHDGQYGDFSDDRDVADGGVSTGT   120
83-211         DKGARFALMFNTNSETELGTLGTYTQLRFNYTSNNSRHDGQYGDFSDSVDVADGGVNTGT   120
               **.***** ..*.******** *:* *****::.**  ** **             . **
                    L3      B6     B7          L4                B8        B9
83-211         VMQFAYIQLGGLRVGIDESEFQTFTGYLGDVINDDVISAGTYRTGKISYTFTGGNGFSAV   169
BO1            VMQFAYIQLGGLRVGIDESEFQTFTGYLGDVINDDVISAGTYRTGKISYTFTGGNGFSAV   169
10RB9213       VMQFAYIQLGGLRVGIDESEFQTFTGYLGDVINDDVISAGTYRTGKISYTFTGGNGFSAV   169
141012304      VMEFAYIQLGGLRVGIDESEFQTFTGYLGDVINDDVISAGSYRTGKISYTFTGGNGFSAV   170
09RB8913       VMEFAYIQLGGLRVGIDESEFQTFTGYLGDVINDDVISAGSYRTGKISYTFTGGNGFSAV   170
09RB8471       VMEFAYIQLGGLRVGIDESEFQTFTGYLGDVINDDVISAGSYRTGKISYTFTGGNGFSAV   170
BO2            VMEFAYIQLGGLRVGIDESEFHTFTGYLGDVINDDVISAGSYRTGKISYTFTGGNGFSAV   170
09RB8908       VMEFAYIQLGGLRVGIDESEFHTFTGYLGDVINDDVISAGSYRTGKISYTFTGGNGFSAV   170
10RB9215       VMEFAYIQLGGLRVGIDESEFHTFTGYLGDVINDDVISAGSYRTGKISYTFTGGNGFSAV   170
CCM 4915       VMEFAYIQLGGLRVGIDESEFHTFTGYLGDVINDDVISAGSYRTGKISYTFTGGNGFSAV   170
16M            VMEFAYIQLGGLRVGIDESEFHTFTGYLGDVINDDVISAGSYRTGKISYTFTGGNGFSAV   170
B14/94         VMEFAYIQLGGLRVGIDESEFHTFTGYLGDVINDDVISAGSYRTGKISYTFTGGNGFSAV   170
B14/94         VMEFAYIQLGGLRVGIDESEFHTFTGYLGDVINDDVISAGSYRTGKISYTFTGGNGFSAV   170
F8/08/60       VMEFAYIQLGGLRVGIDESEFHTFTGYLGDVINDDVISAGSYRTGKISYTFTGGNGFSAV   170
F8/08/60       VMEFAYIQLGGLRVGIDESEFHTFTGYLGDVINDDVISAGSYRTGKISYTFTGGNGFSAV   170
76-250         DLQFAYITLGGFKVGIDESEFHTFTGYLGDIINDDVISAGSYRTGKISYTFTGGNGFSAV   180
45/20          VMEFAYIQLGGLRVGIDESEFHTFTGYLGDVINDDVISAGSYRTGKISYTFTGGNGFSAV   170
F60            DLQFAYITLGGFKVGIDESEFHTFTGYLGDVINDDVVAAGSYRTGKISYTFTGGNGFSAV   180
F60            DLQFAYITLGGFKVGIDESEFHTFTGYLGDVINDDVVAAGSYRTGKISYTFTGGNGFSAV   180
16M            DLQFAYITLGGFKVGIDESEFHTFTGYLGDVINDDVVAAGSYRTGKIAYTFTGGNGFSAV   180
CCM 4915       DLQFAYITLGGFKVGIDESEFHTFTGYLGDVINDDVVAAGSYRTGKIAYTFTGGNGFSAV   180
83-211         DLQFAYITLGGFKVGIDESEFHTFTGYLGDVINDDVVAAGSYRTGKIAYTFTGGNGFSAV   180
                ::**** ***::********:********:*****::**:******:************
```

```
                  B9             L5              B10                  B11       L6
83-211            IALEQGGDNDGGYTPVFKDSQGREINGRGYQIDGYMPDVVGGLKYAGGWGSIAGVVAYDS    229
BO1               IALEQGGDNDGGYTPVFRDSQGNEISGQGYQIDGYMPDVVGGLKYAGGWGSIAGVVAYDS    229
10RB9213          IALEQGGDNDGGYTPTLKE-------GQGYQIDGYMPDVVGGLKYAGGWGSIAGVVAYDS    222
141012304         IALEQGGDNDGGYTG-----------STNYHIDGYMPDVVGGLKYAGGWGSIAGVVAYDS    219
09RB8913          IALEQGGDNDGGYTG-----------STNYHIDGYMPDVVGGLKYAGGWGSIAGVVAYDS    219
09RB8471          IALEQGGDNDGGYTG-----------STNYHIDGYMPDVVGGLKYAGGWGSIAGVVAYDS    219
BO2               IALEQGGDNDGGYTG-----------STNYHIDGYMPDVVGGLKYAGGWGSIAGVVAYDS    219
09RB8908          IALEQGGDNDGGYTG-----------STNYHIDGYMPDVVGGLKYAGGWGSIAGVVAYDS    219
10RB9215          IALEQGGDNDGGYTG-----------TTNYHIDGYMPDVVGGLKYAGGWGSIAGVVAYDS    219
CCM 4915          IALEQGGDNDGGYTG-----------TTNYHIDGYMPDVVGGLKYAGGWGSIAGVVAYDS    219
16M               IALEQGGDNDGGYTG-----------TTNYHIDGYMPDVVGGLKYAGGWGSIAGVVAYDS    219
B14/94            IALEQGGDNDGGYTG-----------ATNCHIDGYMPDVVGGLKYAGGWGSIAGVVAYDS    219
B14/94            IALEQGGDNDGGYTG-----------ATNCHIDGYMPDVVGGLKYAGGWGSIAGVVAYDS    219
F8/08/60          IALEQGGDNDGGYTG-----------TTNYHIDGYMPDVVGGLKYAGGWGSIAGVVAYDS    219
F8/08/60          IALEQGGDNDGGYTG-----------TTNYHIDGYMPDVVGGLKYAGGWGSIAGVVAYDS    219
76-250            IALEQGGDNDGGYTG-----------TTNYHIDGYMPDVVGGLKYAGGWGSIAGVVAYDS    229
45/20             IALEQGGEDV----------------DNDYTIDGYMPHVVGGLKYAGGWGSIAGVVAYDS    214
F60               IALEQGGEDV----------------DNDYTIDGYMPHVVGGLKYAGGWGSIAGVVAYDS    224
F60               IALEQGGEDV----------------DNDYTIDGYMPHVVGGLKYAGGWGSIAGVVAYDS    224
16M               IALEQGGEDV----------------DNDYTIDGYMPHVVGGLKYAGGWGSIAGVVAYDS    224
CCM 4915          IALEQGGEDV----------------DNDYTIDGYMPHVVGGLKYAGGWGSIAGVVAYDS    224
83-211            IALEQGGEDV----------------DNDYTIDGYMPHVVGGLKYAGGWGSIAGVVAYDS    224
                  *******::                    . ******.**********************

                          L6           B12                      B13       L7
83-211            VIEEWATKVRGDVNITDQFSVWLQGAYSSAATPDQNYGQWGGDWAVWGGLKYQATQKAAF    289
BO1               VIEEWATKVRGDVNITDQFSVWLQGAYSSAATPDQNYGQWGGDWAVWGGLKYQATQKAAF    289
10RB9213          VIEEWATKVRGDVNITDRFSVWLQGAYSSAATPDQNYGQWGGDWAVWGGLKYQATQKAAF    282
141012304         VIEEWATKVRGDVNITDQFSVWLQGAYSSAATPNQNYGQWGGDWAVWGGLKYQATQKAAF    279
09RB8913          VIEEWATKVRGDVNITDQFSVWLQGAYSSAATPDQNYGQWGGDWAVWGGLKYQATQKAAF    279
09RB8471          VIEEWATKVRGDVNITDQFSVWLQGAYSSAATPDQNYGQWGGDWAVWGGLKYQATQKAAF    279
BO2               VIEEWATKVRGDVNITDQFSVWLQGAYSSDATPDQNYGQWGGDWAVWGGLKYQATQKAAF    279
09RB8908          VIEEWAAKVRGDVNITDQFSVWLQGAYSSAATPDQNYGQWGGDWAVWGGLKYQATQKAAF    279
10RB9215          VIEEWAAKVRGDVNITDQFSVWLQGAYSSAATPDQNYGQWGGDWAVWGGLKYQATQKAAF    279
CCM 4915          VIEEWAAKVRGDVNITDRFSVWLQGAYSSAATPDQNYGQWGGDWAVWGGLKYQATQKAAF    279
16M               VIEEWAAKVRGDVNITDQFSVWLQGAYSSAATPDQNYGQWGGDWAVWGGLKYQATQKAAF    279
B14/94            VIEEWAAKVRGDVNITDQFSVWLQGAYSSAATPDQNYGQWGGDWAVWGGAKFIATEKATF    279
B14/94            VIEEWAAKVRGDVNITDQFSVWLQGAYSSAATPDQNYGQWGGDWAVWGGAKFIATEKATF    279
F8/08/60          VIEEWAAKVRGDVNITDQFSVWLQGAYSSAATPDQNYGQWGGDWAVWGGLKYQATQKAAF    279
F8/08/60          VIEEWAAKVRGDVNITDQFSVWLQGAYSSAATPDQNYGQWGGDWAVWGGLKYQATQKAAF    279
76-250            VIEEWAAKVRGDVNITDQFSVWLQGAYSSAATPDQNYGQWGGDWAVWGGLKYQATQKAAF    289
45/20             VIEEWAAKVRGDVNITDQFSVWLQGAYSSAATPDQNYGQWGGDWAVWGGLKYQATQKAAF    274
F60               VIEEWATKVRGDVNITDRFSVWLQGVYSSAATPNQNYGQWGGDWAVWGGAKFIATEKATF    284
F60               VIEEWATKVRGDVNITDRFSVWLQGVYSSAATPNQNYGQWGGDWAVWGGAKFIATEKATF    284
16M               VIEEWATKVRGDVNITDRFSVWLQGAYSSAATPNQNYGQWGGDWAVWGGAKFIATEKATF    284
CCM 4915          VIEEWATKVRGDVNITDRFSVWLQGAYSSAATPNQNYGQWGGDWAVWGGAKFIATEKATF    284
83-211            VIEEWATKVRGDVNITDRFSVWLQGAYSSAATPNQNYGQWGGDWAVWGGAKFIATEKATF    284
                  ******:**********:*******.*** ***:*************** *: **:**:*

                       L7          B14          B15          L8            B16
83-211            NLQAAHDDWGKTAVTANVAYELVPGFTITPEVSYTKFSNEWKRELGNDTLDDAWGGIVRF    349
BO1               NLQAAHDDWGKTAVTANVAYELVPGFTITPEVSYTKFSNEWKRELGNDTLDDAWGGIVRF    349
10RB9213          NLQAAHDDWGKTAVTANVAYELVPGFTITPEVSYTKFSNEWKRELANDTLDDAWGGIVRF    342
141012304         NLQAAHDDWGKTAVTANVAYELVPGFTITPEVSYTKFSNEWKRQLANDTLDDAWGGIVRF    339
09RB8913          NLQAAHDDWGKTAVTANVAYELVPGFTITPEVSYTKFSNEWKRQLANDTLDDAWGGIVRF    339
09RB8471          NLQAAHDDWGKTAVTANVAYELVPGFTITPEVSYTKFSNEWKRQLANDTLDDAWGGIVRF    339
BO2               NLQAAHDDWGKTAVTANVAYELVPGFTITPEVSYTKFSNEWKRQLANDTLDDAWGGIVRF    339
09RB8908          NLQAAHDDWGKTAVTANVAYELVPGFTVTPEVSYTKFGGEWKNTVAE---DNAWGGIVRF    336
10RB9215          NLQAAHDDWGKTAVTANVAYELVPGFTVTPEVSYTKFGGEWKNTVAE---DNAWGGIVRF    336
CCM 4915          NLQAAHDDWGKTAVTANVAYELVPGFTVTPEVSYTKFGGEWKNTVAE---DNAWGGIVRF    336
16M               NLQAAHDDWGKTAVTANVAYELVPGFTVTPEVSYTKFGGEWKNTVAE---DNAWGGIVRF    336
B14/94            NLQAAHDDWGKTAVTANVAYQLVPGFTITPEVSYTKFGGEWKDTVAE---DNAWGGIVRF    336
B14/94            NLQAAHDDWGKTAVTANVAYQLVPGFTITPEVSYTKFGGEWKDTVAE---DNAWGGIVRF    336
F8/08/60          NLQAAHDDWGKTAVTANVAYELVPGFTVTPEVSYTKFGGEWKDTVAE---DNAWGGIVRF    336
F8/08/60          NLQAAHDDWGKTAVTANVAYELVPGFTVTPEVSYTKFGGEWKDTVAE---DNAWGGIVRF    336
76-250            NLQAAHDDWGKTAVTANVAYELVPGFTVTPEVSYTKFGGEWKNTVAE---DNAWGGIVHF    346
45/20             NLQAAHDDWGKTAVTANVAYELVPGFTVTPEVSYTKFGGEWKNTVAE---DNAWGGIVRF    331
F60               NLQAAHDDWGKTAVTANVAYELVPGFTVTPEVSYTKFGGEWKNTVAE---DNAWGGIVRF    341
F60               NLQAAHDDWGKTAVTANVAYELVPGFTVTPEVSYSKFGGEWKDTVAE---DNAWGGIVRF    341
16M               NLQAAHDDWGKTAVTANVAYQLVPGFTITPEVSYTKFGGEWKDTVAE---DNAWGGIVPF    341
CCM 4915          NLQAAHDDWGKTAVTANVAYQLVPGFTITPEVSYTKFGGEWKDTVAE---DNAWGGIVRF    341
83-211            NLQAAHDDWGKTAVTANVAYQLVPGFTITPEVSYTKFGGEWKDTVAE---DNAWGGIVRF    341
                  ********************:******:******:**..***  :.:   *:****** *
```

```
                 B16
83-211           QRSF 353
BO1              QRSF 353
10RB9213         QRSF 346
141012304        QRSF 343
09RB8913         QRSF 343
09RB8471         QRSF 343
BO2              QRSF 343
09RB8908         QRSF 340
10RB9215         QRSF 340
CCM 4915         QRSF 340
16M              QRSF 340
B14/94           QRSF 340
B14/94           QRSF 340
F8/08/60         QRSF 340
F8/08/60         QRSF 340
76-250           QRSF 350
45/20            QRSF 335
F60              QRSF 345
F60              QRSF 345
16M              QRSF 345
CCM 4915         QRSF 345
83-211           QRSF 345
                 ****
```

Supplementary Figure S3. Multiple amino acid sequence alignment of Omp2a and Omp2b proteins, from representative *Brucella* strains containing insertions or deletions in the respective gene. The strains are indicated on the left, when Omp2a is used the corresponding strain number is highlighted in yellow. The predicted surface loops are highlighted in blue, according to Paquet et al. (2001). Underlined sequences indicate the predicted flanking transmembrane β-strands (B1 to B16) (Paquet et al., 2001). Omp2a-specific amino acids are highlighted in yellow, in accordance with the Omp2a and Omp2b reference sequence used from *Brucella* sp. 83-211. Omp2 amino acids colored in red are independent from gene conversion according to the same reference sequences.
